# Spin Hall Nano‐Antenna

**DOI:** 10.1002/advs.202520505

**Published:** 2026-02-25

**Authors:** Raisa Fabiha, Pratap Kumar Pal, Michael Suche, Amrit Kumar Mondal, Erdem Topsakal, Anjan Barman, Supriyo Bandyopadhyay

**Affiliations:** ^1^ Department of Electrical and Computer Engineering Virginia Commonwealth University Richmond Virginia USA; ^2^ Department of Condensed Matter and Materials Physics S. N. Bose National Centre for Basic Sciences Kolkata India; ^3^ Department of Physics, School of Natural Sciences Shiv Nadar Institution of Eminence Dadri Uttar Pradesh India

**Keywords:** inverse spin Hall effect, magnon–photon coupling, nano‐antenna, spin Hall effect, spin pumping, spin–orbit torque

## Abstract

The spin Hall effect is a well‐known phenomenon in spintronics that has found numerous applications in digital electronics (memory and logic), but relatively few in analog electronics. Practically the only analog application in widespread use is the spin Hall nano‐oscillator (SHNO) that delivers a high frequency alternating current or voltage to a load. Here, we report its analogue – a spin Hall nano‐antenna (SHNA) that radiates a high frequency electromagnetic wave into the surrounding medium. It can also radiate an acoustic wave into an underlying substrate if the nanomagnets are made of a magnetostrictive material. That makes it a *dual* electromagnetic/acoustic antenna. The SHNA is made of an array of ledged magnetostrictive nanomagnets deposited on a substrate, with a heavy metal nanostrip underlying/overlying the ledges. An alternating charge current passed through the nanostrip generates an alternating spin–orbit torque (SOT) in the nanomagnets via the spin Hall effect, which makes their magnetizations oscillate in time with the frequency of the current, producing confined spin waves (magnons) in the nanomagnets. These spin waves radiate electromagnetic waves (photons) in space owing to the transfer of energy from magnons to photons, thereby implementing a *transmitting* antenna. Curiously, although the SHNA is much smaller in size than the radiated electromagnetic wavelength and hence qualifies as a “point source”, it does *not* radiate isotropically owing to the anisotropy of the (frequency‐dependent) spin wave patterns that form within the odd‐shaped nanomagnets, which endows the “point source” with internal anisotropy.

The same device can also act as a *receiving* antenna. Incident electromagnetic radiation generates polychromatic spin waves in the nanomagnets, which pump spin into the heavy metal nanostrips at their own frequencies, giving rise to a polychromatic alternating voltage across the latter owing to the ac inverse spin Hall effect. This implements the receiver. The transmitting/receiving gain and radiation efficiency of the SHNA far exceed those of a conventional antenna of the same size, thereby enabling extreme antenna miniaturization.

## Graphical abstract

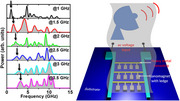

## 1 Introduction

The spin Hall effect [1, 2] is a remarkable phenomenon in spintronics that is used to inject spin currents into ferromagnets by passing charge currents through an underlying/overlying heavy metal or topological insulator [3, 4]. This results in a spin–orbit torque (SOT) within the ferromagnet, which is harnessed to switch its magnetization, thereby making it an efficient mechanism to write bits into non‐volatile magnetic memory [5, 6]. The same effect has also been leveraged to implement combinational Boolean logic using magnetic switches [7]. These are all digital applications. Analog applications of the spin Hall effect are few and far between, with the most notable application being in the spin Hall nano‐oscillator (SHNO) [8] which is used in microwave assisted magnetic recording [9], neuromorphic computing [8], etc.

**FIGURE 1:**
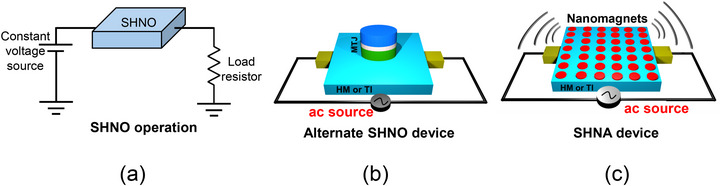
(a) A spin Hall nano‐oscillator (SHNO) acts as a time‐varying resistor that delivers a time‐varying current to a load resistor connected in series with it when both are powered by a dc voltage source. (b) Schematic of a SHNO device actuated by passing an alternating current through a heavy metal (HM) or topological insulator (TI) and no external magnetic field present. (c) Schematic of a spin Hall nano antenna (SHNA) where, again, no external magnetic field is needed.

SHNOs are oscillators that deliver an alternating current to a load resistor [8] as shown in Figure 1a. The device usually consists of a magnetic tunnel junction (MTJ) whose soft layer is placed in contact with a heavy metal layer (HM) or a topological insulator (TI). The MTJ is actually not required for a SHNO, but provides a convenient load and spin‐to‐charge converter. An external magnetic field (or some other stimulus that emulates the effect of a magnetic field) sets up magnetization precession in the soft layer to produce spin waves, and the precession is sustained by passing a direct current through the HM or TI, which generates an anti‐damping spin–orbit torque on the soft layer of the MTJ via the spin Hall effect. This torque counters any damping and sustains the oscillations in time. Because the magnetization of the soft layer oscillates in time, the MTJ acts as an oscillating resistor that will deliver a time varying current to a load resistor when both are placed in series with a constant voltage source (see Figure 1a).

The disadvantage of this approach is the need for an external magnetic field (or something else that *emulates* a magnetic field) [10] to set up the magnetization precession or spin waves. True field‐free oscillators, working on a somewhat different principle (they are not a SHNO), have been proposed [11], but not yet experimentally demonstrated. We can also drive an alternating (instead of direct) current through the HM or TI (see Figure 1b) to generate an *alternating* (instead of time‐invariant) spin–orbit torque in the soft layer. That will induce either back‐and‐forth domain wall motion [12] or magnetization precession [13] to generate spin waves (magnetization oscillations) in the soft layer, predominantly at the frequency of the ac current, and without the need for any magnetic field. The MTJ will then once again act as an oscillating resistor (because the soft layer's magnetization is oscillating) which will deliver an alternating current to a load with the frequency of the injected current. This is, of course, not a true SHNO since we are not converting dc power from a dc current/voltage to ac power, which an “oscillator” should, but it will nonetheless produce the oscillating resistor and hence an oscillating current/voltage delivered to a load – this time without the need for an external magnetic field.

The magnetization oscillation generated by an alternating spin–orbit torque may have other uses as well that are very distinct from an oscillator. Here, we have leveraged it to implement an “antenna” that radiates electromagnetic waves into the surrounding medium with the frequency of the injected alternating current. The fact that an alternating spin–orbit torque generated by passing an alternating current through a HM layer produces magnetization oscillation (spin wave) of the same frequency in a nanomagnet in contact with the HM, has been verified experimentally by us in the past [14]. It has also been validated theoretically [13].

An *antenna* is very distinct from an *oscillator*. While the former delivers an ac current or voltage to a load resistor, the latter “radiates” an ac signal (electromagnetic wave) into the surrounding medium, as shown in Figure 1c. There are other differences as well. The oscillator outputs only one fixed frequency determined by its parameters (e.g., the magnetic field in the case of the SHNO) while an antenna can output *any* frequency that is the frequency of the input signal. The standard definition of an oscillator is a device that intakes dc power and outputs ac power, which is exactly what the SHNO does. The intake is the dc spin–orbit torque, and the output is an ac signal of fixed frequency that is determined by the applied magnetic field. We can of course change the frequency by changing the magnetic field, but at any given field, the frequency is fixed. The standard definition of an antenna, in contrast, is that intakes an ac signal of variable frequency and radiates an ac signal of the same variable frequency into space.

Various types of modulation schemes (frequency modulation, phase‐shift keying, frequency‐shift keying, etc.) of spin torque (and by extension, spin Hall) nano‐oscillators have been demonstrated for signal processing [15, 16, 17, 18, 19, 20], but there has been no demonstration of antenna functionality (wireless signal transmission over long distances). Here, we report a **spin Hall nano‐antenna (SHNA)** made of nanomagnets placed in contact with a HM nanostrip through which an ac current is made to flow. The resulting alternating spin–orbit torque sets up magnetization oscillations (confined spin waves) in the nanomagnets at the frequency of the current. These spin waves transfer their energy to electromagnetic waves via magnon–photon coupling to radiate an electromagnetic wave into the surrounding medium at the frequency of the ac current, thereby implementing an antenna. In Section S12, we have provided a classical phenomenological theory of how alternating spin–orbit torque can produce electromagnetic radiation. This theory is a classical theory based on coupled Maxwell's equation and Landau–Lifshitz–Gilbert equation. It provides a phenomenological explanation of the antenna principle.

The SHNA that we have demonstrated is an extreme sub‐wavelength antenna whose dimensions (sub‐mm) are more than an order of magnitude smaller than the radiated electromagnetic wavelength at the frequencies we have tested, which are between 1 and 10 GHz (free space wavelength 3–30 cm), and yet it radiates efficiently. Had it been a conventional antenna, its radiation efficiency would have been very poor [21, 22, 23]. This is an entirely new application of spin–orbit torque and spin Hall effect, and it can spawn a new genre of ultra‐miniaturized transmitting antennas.

We point out that recently there have been some reports of other spintronic nano‐antennas (e.g., [24, 25]) which radiate spin waves (not electromagnetic waves) as well as those that radiate electromagnetic waves due to transfer of energy from spin waves into electromagnetic waves [26, 27, 28, 29]. However, in those latter antennas, the spin waves are generated by acoustic waves via magnon–phonon (magneto–elastic) coupling and *not* by the Spin Hall Effect or spin–orbit torque. That differentiates this work from previous work in “nanomagnetic antennas”. The antenna reported here has an advantage. Those based on magneto–elastic coupling would require a piezoelectric substrate to generate the acoustic waves, whereas here we do not need such a substrate and can use silicon, which makes it compatible with silicon technology that dominates electronics. Consequently, our SHNA can be easily integrated onto a silicon chip for on‐chip communication.

## 2 Transmitting Antenna

### 2.1 Sample Description

**FIGURE 2:**
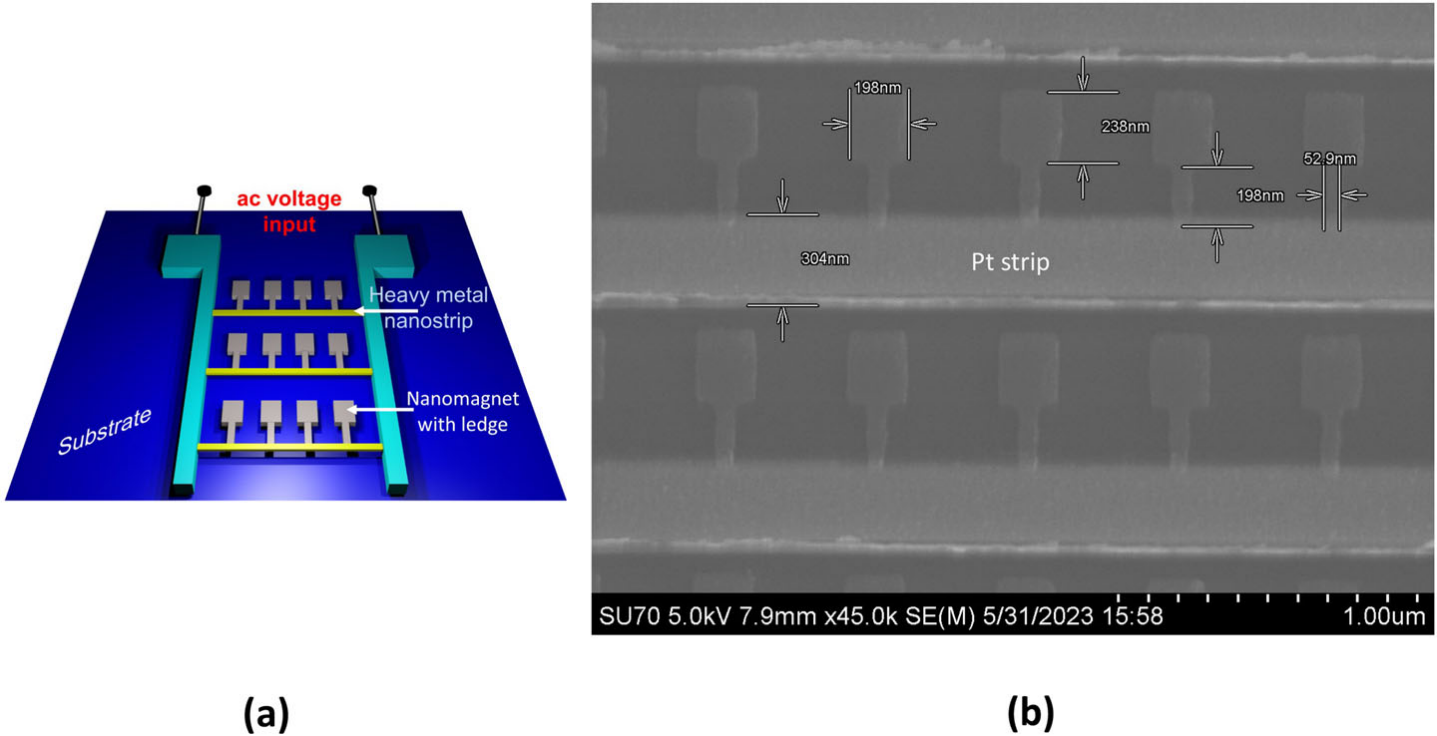
(a) Schematic of the spin Hall nano‐antenna device. (b) Scanning electron micrograph of the device showing the various structural dimensions).

The schematic of our SHNA device is shown in Figure 2a. It consists of linear periodic arrays of “ledged” rectangular nanomagnets deposited on a ${\mathrm{LiNbO}}_{3}$ substrate, with a Pt nanostrip underlying the ledges, as shown in Figure 2. The reason why we use ${\mathrm{LiNbO}}_{3}$ instead of Si will be clear later; we can make our SHNA act as a dual electromagnetic and acoustic antenna and the acoustic functionality requires a piezoelectric substrate. Had we been interested in only an electromagnetic antenna, we could have used a Si substrate.

The Pt nanostrip is ∼300 nm wide and 5 nm thick. There are 3000 linear arrays, each containing 95 nanomagnets (total of 285 000 nanomagnets), and the ends of the nanostrips in each array are connected to two contact pads as shown in Figure 2a. Alternating charge current is passed between these two pads to generate alternating spin–orbit torques on the nanomagnets, which produce spin waves that radiate electromagnetic waves. The nanomagnets are made of Co, a magnetostrictive ferromagnet, and their thickness is 15 nm. Nanomagnet and ledge dimensions, intermagnet separation, etc. are all shown in the scanning electron micrograph in Figure 2b. The inter‐nanomagnet separation is large enough that any dipole interaction between neighbors is negligible.

### 2.2 Transmitting Antenna Activation

An alternating charge current is pumped into each Pt nanostrip, which injects spin currents of alternating spin polarization into the nanomagnets via the spin Hall effect [1, 2]. This causes an alternating spin–orbit torque [31] that results in either back‐and‐forth domain wall motion in the nanomagnets [12], or magnetization precession [13], or both. That excites spin waves within the nanomagnets [13, 14]. The spin waves transfer their energy to electromagnetic waves, which are radiated in the surrounding medium, thereby implementing transmitting antenna action.

The efficiency of electromagnetic wave generation by spin waves depends on magnon–photon coupling, which is usually weak, but could be strong in some circumstances [32, 33]. Notably, it has been shown recently that interfacial spin–orbit torque at the interface of a ferromagnet and a heavy metal (exactly our case) can make magnon–photon coupling much stronger than usual [34]. Ref. [34] showed the generation of magnons by photons due to magnon–photon coupling. Here, we are demonstrating the generation of photons by magnons. Mode coupling is generally a reciprocal phenomenon. If mode A couples into mode B and transfers its energy to mode B, then mode B can also transfer its energy to mode A via coupling. In our case, spin wave modes in the nanomagnets generated by the alternating spin–orbit torque transfer their energy to radiative electromagnetic modes and produce the radiation.

#### 2.2.1 Why the Nanomagnets Have Ledges – Dual Electromagnetic and Acoustic Antennas

There is a reason why the nanomagnets are ledged, and the Pt strip is placed only over the ledges. Since Co is magnetostrictive, it physically expands and contracts when its magnetization alternates with the frequency of the pumped ac current. If we place the Pt strip directly on the nanomagnets to generate the alternating spin–orbit torque, it will “clamp” the nanomagnets and prevent the expansion/contraction, which will quench the spin waves and encumber the radiation process. It is imperative to *not* clamp the nanomagnets if they are magnetostrictive. This is the reason that the Pt strip is placed only on the ledges so as to not encumber the expansion/contraction of the bulk of the nanomagnets. The bottom surfaces of the nanomagnets are of course clamped by the underlying substrate, but this does not hinder the expansion/contraction of the top layers, and hence does not prevent the generation of confined spin waves within the nanomagnets, which radiate electromagnetic waves.

Of course, the ledges would have been entirely unnecessary if we had chosen a non‐magnetostrictive or weakly magnetostrictive ferromagnet like Fe (instead of Co), which would not have experienced the expansion/contraction. We did not do that for a reason. If we can make the nanomagnets expand and contract with the frequency of the ac current (which requires sufficient magnetostriction), then this expansion/contraction will generate a time‐varying strain in the substrate underneath, which will cause a surface acoustic wave (SAW) of the same frequency as the ac current to propagate in the substrate. That SAW can be picked up with interdigitated transducers if the substrate is piezoelectric (this is the reason for using the ${\mathrm{LiNbO}}_{3}$ substrate; otherwise Si would work just as well). This will make this construct act as a *dual electromagnetic and acoustic antenna* that radiates an electromagnetic wave into space while simultaneously radiating an acoustic wave in the underlying substrate. The acoustic antenna functionality was already demonstrated by us in the past [14] and hence not addressed here.

We note that magnetostrictive nanomagnets and a piezoelectric substrate will be needed *only* if we wish to implement a *dual* electromagnetic and acoustic antenna. If only an electromagnetic antenna is needed, we do not need either magnetostrictive nanomagnets or a piezoelectric substrate.

### 2.3 Spin Waves in the Nanomagnets Due to Alternating Spin Hall Effect Caused by the Alternating Current

**FIGURE 3:**
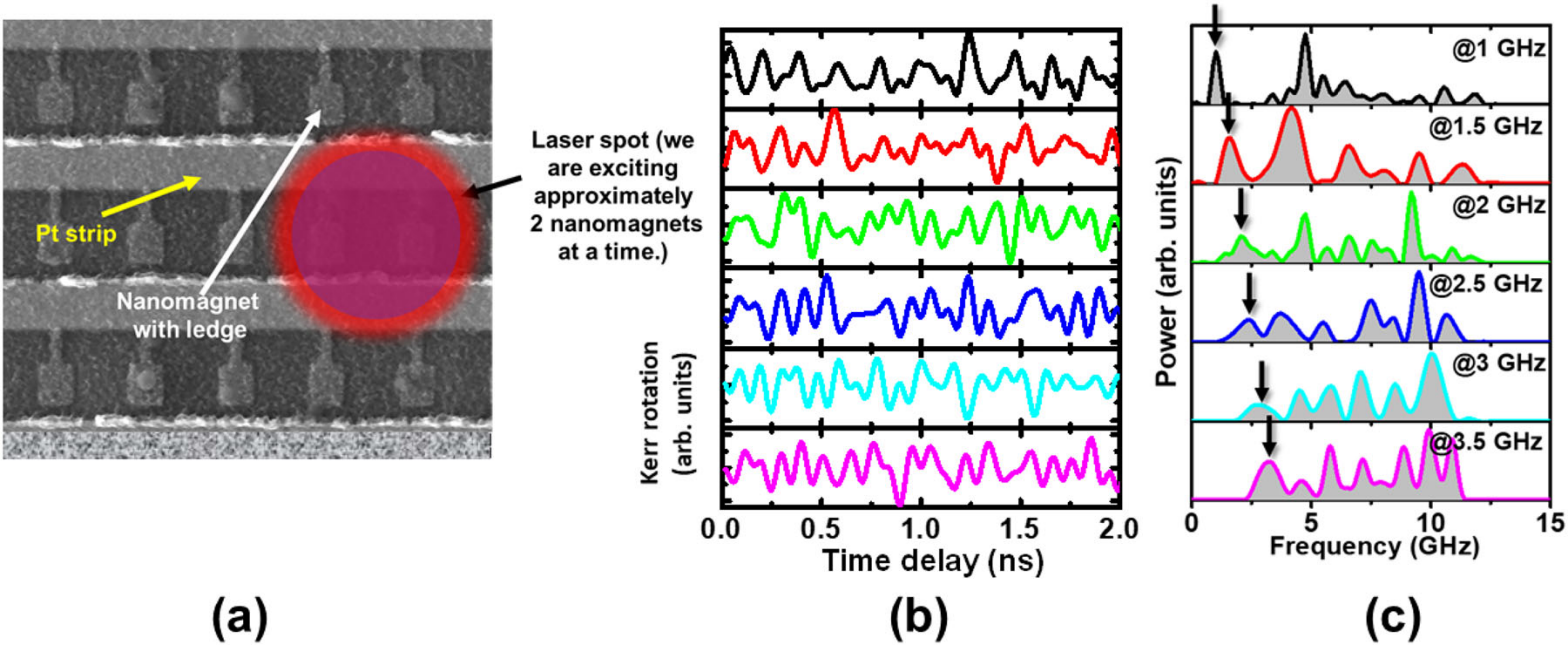
(a) The pump and probe laser spots are approximately 1 μm in diameter and hence cover two nanomagnets at a time. Hence, the spin waves are always sampled from two nanomagnets. (b) Kerr oscillations in the nanomagnets plotted in the time domain. (c) Fast Fourier transform of the Kerr oscillations showing the peaks in the spin wave spectra.

To further ascertain that pumping an alternating current into the heavy metal nanostrip over(under)lying the ledges of the nanomagnets indeed excites spin waves (magnons) in them, we carried out time‐resolved magneto‐optical Kerr effect (TR‐MOKE) measurements to confirm that spin waves are generated in the nanomagnets, and then proceeded to find their frequency spectra. The details of the TR‐MOKE setup are provided in Section S1. We pumped alternating current into the heavy metal nanostrip at six different frequencies of 1, 1.5, 2, 2.5, 3 and 3.5 GHz with constant input power of 16 dbm. The pump and probe beam laser spots of the TR‐MOKE setup overlap in space and cover only two nanomagnets at a time, and hence we are sampling the spin waves from only two nanomagnets, as shown in Figure 3a. The measured Kerr‐oscillations are shown in Figure 3b. Fast Fourier transform of these oscillations reveals the peaks in the spectra of the spin waves excited by the ac current, which are shown in Figure 3c. It is interesting to note that while there is always a peak at the current pumping frequency (as expected), there are also other peaks at higher frequencies which are not necessarily integral multiples of the pumping frequencies. These other peaks are obviously not associated with the ac current injection since they occur at arbitrary frequencies. Further investigation showed that the frequencies where these extra peaks occur vary from one region of the nanomagnet array to another. Recall that the pump and probe beam of the TR‐MOKE setup samples only two nanomagnets at a time; hence, we are always probing the modes locally, i.e. within those two nanomagnets. If we focus the pump and probe beam on a *different* nanomagnet pair, then the mode at the ac current frequency remains unchanged in frequency, but the other mode frequencies change. This is shown in Section S4. Thus, the higher frequency modes are different in different regions of the nanomagnet array and ensemble averaging over the entire array will wash them out. Therefore, we do not expect to see electromagnetic radiation emanating at these higher frequencies, but instead expect to see radiation only at the frequency of the injected ac current since ensemble averaging over the entire nanomagnet array does not wash that out.

We cannot confirm the origin of the spatially‐varying high frequency modes conclusively, but very likely they are vortex modes caused by strain pulses generated in the nanomagnet by the laser heating and cooling. The heating and cooling by the pump and probe pulses will cause strain pulses in the nanomagnets because of the unequal thermal expansion coefficients of the nanomagnets and the substrate [35, 36, 37]. It has been shown that such strain pulses spawn vortex modes in magnetostrictive nanomagnets [40] and that the spectra of these modes depend on the nanomagnet diameter (D) and thickness (t). Since both D and t vary somewhat across the nanomagnet array, we expect to see variance in the frequencies of the high frequency modes, and that is exactly what we see. Because of this variance, if we ensemble average the high frequency modes across the entire nanomagnet array of 285 000 nanomagnets that are present in our samples, their amplitudes will become negligible compared to that of the one occurring at the frequency of the injected ac current. As a result, we cannot observe any electromagnetic radiation with the frequencies of the spatially‐varying high‐frequency modes, but can only observe electromagnetic radiation at the alternating current frequency.

### 2.4 Electromagnetic Radiation Spectrum

**FIGURE 4:**
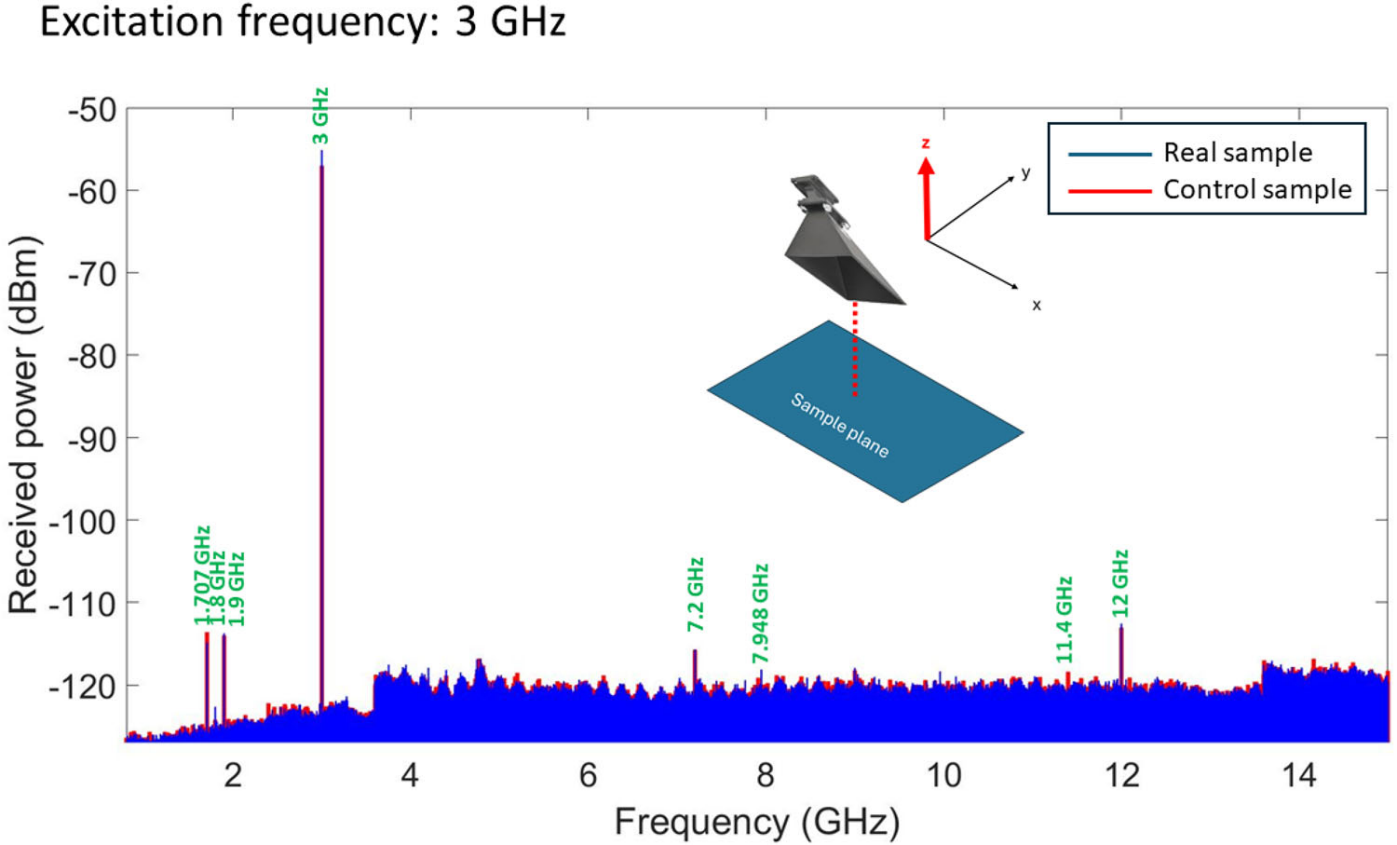
Electromagnetic radiation spectrum of the real and control samples when the frequency of the ac current pumped into the Pt nanostrip is set to 3 GHz and the input power is 15 dbm. The distance between the receiving horn antenna and the sample in this case is 81 cm which is 8 times the wavelength, ensuring that we are measuring the far‐field radiation.

We measured the spectra of the electromagnetic radiation emitted by the samples in an AMS‐5701 anechoic chamber, with the detecting horn antenna facing the plane of the nanomagnets as shown in the inset of Figure 4. A spectrum analyzer was connected to the horn antenna to measure the spectrum of the received emission. The sample was placed at a distance of 81 cm from the horn antenna to ensure that we are always measuring the far‐field radiation at the excitation frequency. The input power from the ac current source was set to 15 dbm (31 mW).

We had also measured the spectrum of the scattering parameter $S_{11}$ with a vector network analyzer, and that data are shown in Section S9. There are two sharp notches in the $S_{11}$ spectrum at 3.1 and 5.8 GHz, showing stronger coupling from the alternating current source into the sample at these frequencies, possibly because of better impedance matching at these two frequencies. Because of this, we measured the spectrum of the emitted radiation with the driving alternating current frequency set to 3 GHz.

The radiation measured by the horn antenna in the anechoic chamber is of course not solely due to the nanomagnets emitting, but also has contributions from the Pt nanostrip, the contact lines, the contact pads and any other extraneous source of radiation. In an attempt to separate out the extraneous contributions, we fabricated two sets of samples that are otherwise nominally identical, except one has nanomagnets and the other does not. We call the latter “control sample” and measure the spectrum of the radiation it emits for comparison with that of the real sample, which contains the nanomagnets. In Figure 4, we show the measured spectra from both the real and the control sample. Screenshots of the spectra taken from the spectrum analyzer can be found in Section S6.

It is important to understand that the difference between the power received at any point in space from the real and the control sample need not be positive because of wave *interference*. The wave emitted from the nanomagnets and that emitted by the peripherals (contact pads, etc.) interfere at any point in space. Destructive interference will make the power received from the real sample (containing nanomagnets plus peripherals) *less* than that received from the control sample (containing only the peripherals), whereas constructive interference will have the opposite effect. This introduces an ambiguity that cannot be resolved easily. Furthermore, in our case, the observed difference is small in magnitude. The power received from the real sample by the horn antenna placed directly above the plane of the nanomagnets was –55 dbm (3.16 nW) whereas that received from the control sample was –57.5 dbm (1.77 nW). The difference of 1.4 nW, albeit well above the noise floor in the anechoic chamber [which was –120 dbm (1 pW)], is too small to allow us to make any quantitative estimates with confidence. We also cannot determine how much of this difference actually accrues from the nanomagnets and how much is due to unavoidable slight differences between the peripherals (i.e. contact pads, etc.) in the real sample and control sample. Furthermore, as we show in the next section, the nanomagnets radiate anisotropically, so that the difference between the power received from the real sample and the control sample will be different in different directions for the same source‐detector separation. All this prevents us from drawing any quantitative inferences and hinders us from determining the radiation efficiency of the SHNA. This is unfortunately unavoidable, and there is no remedy. However, we emphasize that there is no ambiguity about the fact that the nanomagnets do radiate electromagnetic waves because the difference between the radiation from the real and the control sample *can be very large in certain directions at certain frequencies*, leaving no doubt that the nanomagnets are efficient antennas. Moreover, they are very *directional* (i.e. anisotropic radiators) because the difference between the powers received from the real and control samples vary strongly with direction. We discuss more of this later.

We also notice in Figure 4 that there are satellite peaks in the radiation spectrum that are present in both the real sample and the control sample. They are much weaker than the main peak. These satellite peaks do *not* conform to the higher frequency spin wave modes observed in Figure 3. Furthermore, the control sample, which does not have any nanomagnets, also emits those peaks. This tells us that these satellite peaks are either from extraneous sources, or generated by the peripherals, and are *not* associated with the vortex modes generated in the nanomagnets by laser heating/cooling. As already mentioned, those vortex modes ensemble average out when averaged over the entire nanomagnet array and hence do not contribute to measurable radiation.

We conclude this section by pointing out that it is well‐known in the context of conventional antennas that the gain, bandwidth and radiation efficiency will plummet if the antenna dimension is shrunk to small fractions of the electromagnetic wavelength that it radiates [21, 22, 23]. However, that happens only if the antenna is actuated by traditional electromagnetic resonance (oscillating electric dipoles). There is a school of thought that believes that if an antenna is actuated by acoustic resonance instead of electromagnetic resonance, then the relevant wavelength will be the acoustic wavelength at the frequency of radiation and not the electromagnetic wavelength. This will allow antennas to be miniaturized to the acoustic wavelength – which is much smaller than the *electromagnetic* wavelength at the same frequency – without sacrificing radiation efficiency or gain [23]. This prompted significant research in acoustically actuated magneto‐electric antennas of various types [26, 27, 28, 29]. Their intrinsic radiation efficiencies did beat the theoretical limit on the radiation efficiencies of conventional antennas actuated by electromagnetic resonance, sometimes even by many orders of magnitude, which lends credence to this idea. The SHNA that is presented here, however, is *not* actuated acoustically and is of a completely different flavor. This new genre of antennas can open a new direction of research in electrically small (miniaturized) antennas whose dimensions are more than an order of magnitude smaller than the electromagnetic wavelength, and yet they radiate efficiently.

### 2.5 Radiation Patterns of the Transmitting Antenna

**FIGURE 5:**
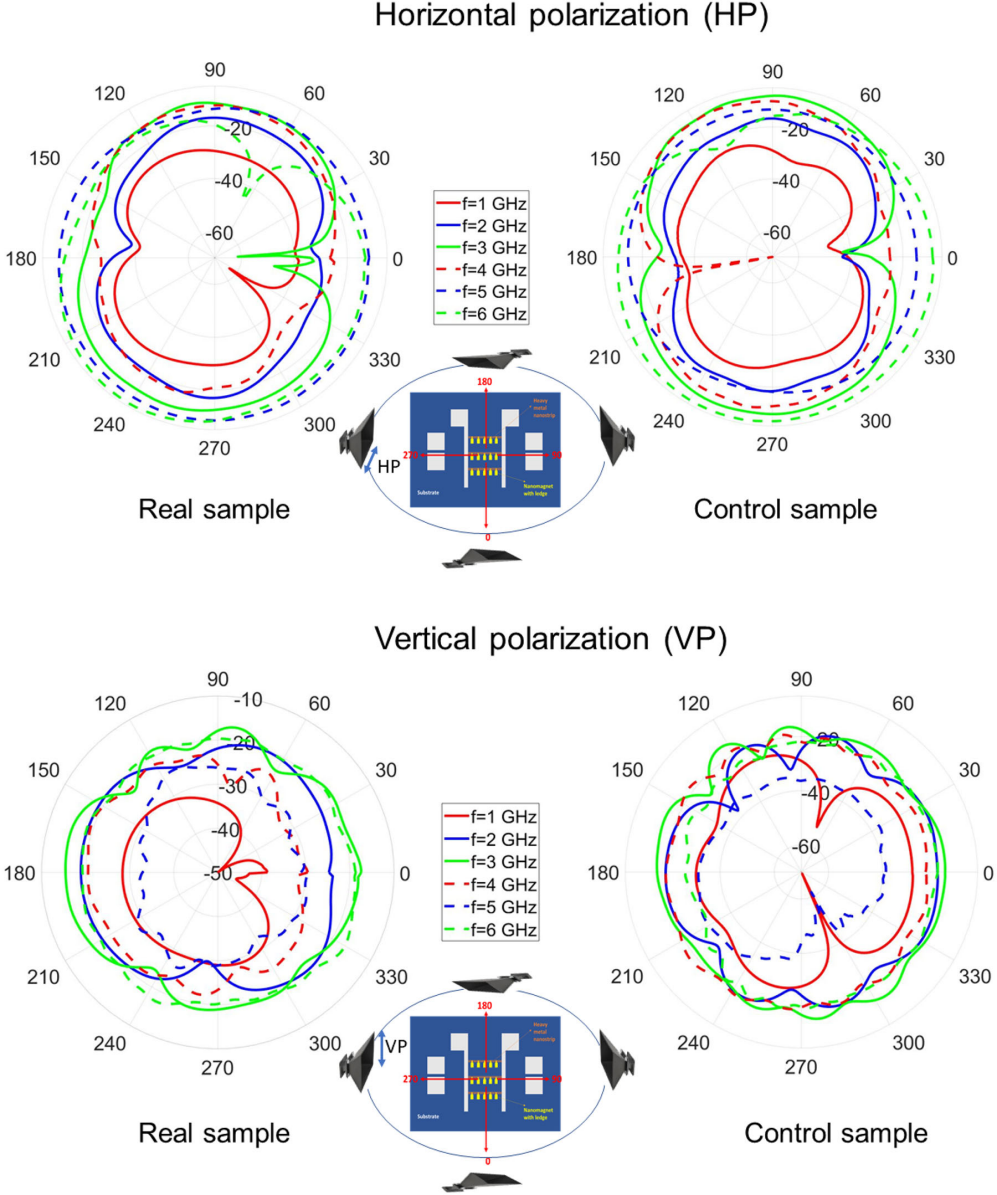
The radiation pattern of the SHNA at different frequencies in the plane of the nanomagnets plotted in dbi. The patterns are shown for both the real sample and the control sample, as well as for both horizontal and vertical polarizations.

We measured the radiation patterns of the transmitting SHNA in three different planes – the plane of the nanomagnets and the two transverse planes. They were measured at frequencies of 1, 2, 3, 4, 5 and 6 GHz (wavelengths 5–30 cm). The detector was always placed at a distance of 81 cm from the sample, which means that we are detecting the far‐field radiation pattern at all frequencies except 1 GHz. The patterns were measured for both the real sample and the control sample. The results are shown in Figure 5 for both horizontal and vertical polarizations in the plane of the nanomagnets. The radiation patterns in the two transverse planes can be found in Section S7.

Since the radiation pattern of the real sample is very different (not just slightly different) from that of the control sample, we can conclude with certainty that the nanomagnets are radiating. What might appear surprising is that in some directions, the received power from the control sample is actually *more* than that from the real sample. This anomaly can be explained by destructive interference. In these cases, the waves transmitted by the peripherals (contact pads, etc.) and the nanomagnets in the real sample interfered destructively where the detector was placed to make the power received from the real sample less than that from the control sample. However, in some directions, the power received from the real sample far exceeds that from the control sample. For example, at 1 GHz frequency and for the vertical polarization, the measured gain in the 290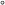 direction in the plane of the nanomagnets exceeds –30 dbi for the real sample and falls short of –60 dbi for the control sample (see Figure 5). This difference of more than 30 dbi (a factor of 1000×) is large enough that it cannot be ascribed to small unavoidable differences between the peripherals (contact pads, etc.) in the two samples. Hence, we can claim with confidence that *the nanomagnets are radiating*, certainly in the 290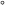 direction at 1 GHz, and other directions as well. This is further confirmatory evidence of the spin Hall nano‐antenna (SHNA) operation.

We note from Figure 5 that the difference between the radiation from the real sample and the control sample varies quite strongly with direction, which means that the nanomagnets are radiating *anisotropically* and in fact the radiation is very “directional” since, for example, it is much stronger in the 290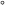 direction than in others. This anisotropy is surprising since the lateral dimension of the entire nanomagnet array (∼ 160 μm) is much smaller than the electromagnetic wavelength at all measurement frequencies. Hence, the entire nanomagnet array could be viewed as a point source that should radiate isotropically. Yet, it does not. The reason for the anisotropy is explained in Section S15. The anisotropy accrues from the anisotropy of the spin wave patterns that form within the nanomagnets. The spin wave patterns are anisotropic because of the odd shapes of the ledged nanomagnets.

We also note that the radiation pattern of the control sample (which has no nanomagnets) is anisotropic as well, but this is to be expected. The radiation pattern of almost any sample that is comparable to or larger than the wavelength is naturally anisotropic, and the control sample is in that category. Hence, we expect its radiation pattern to be anisotropic. This has no bearing on the fact that the radiation from the nanomagnets is anisotropic and strongly directional.

**FIGURE 6:**
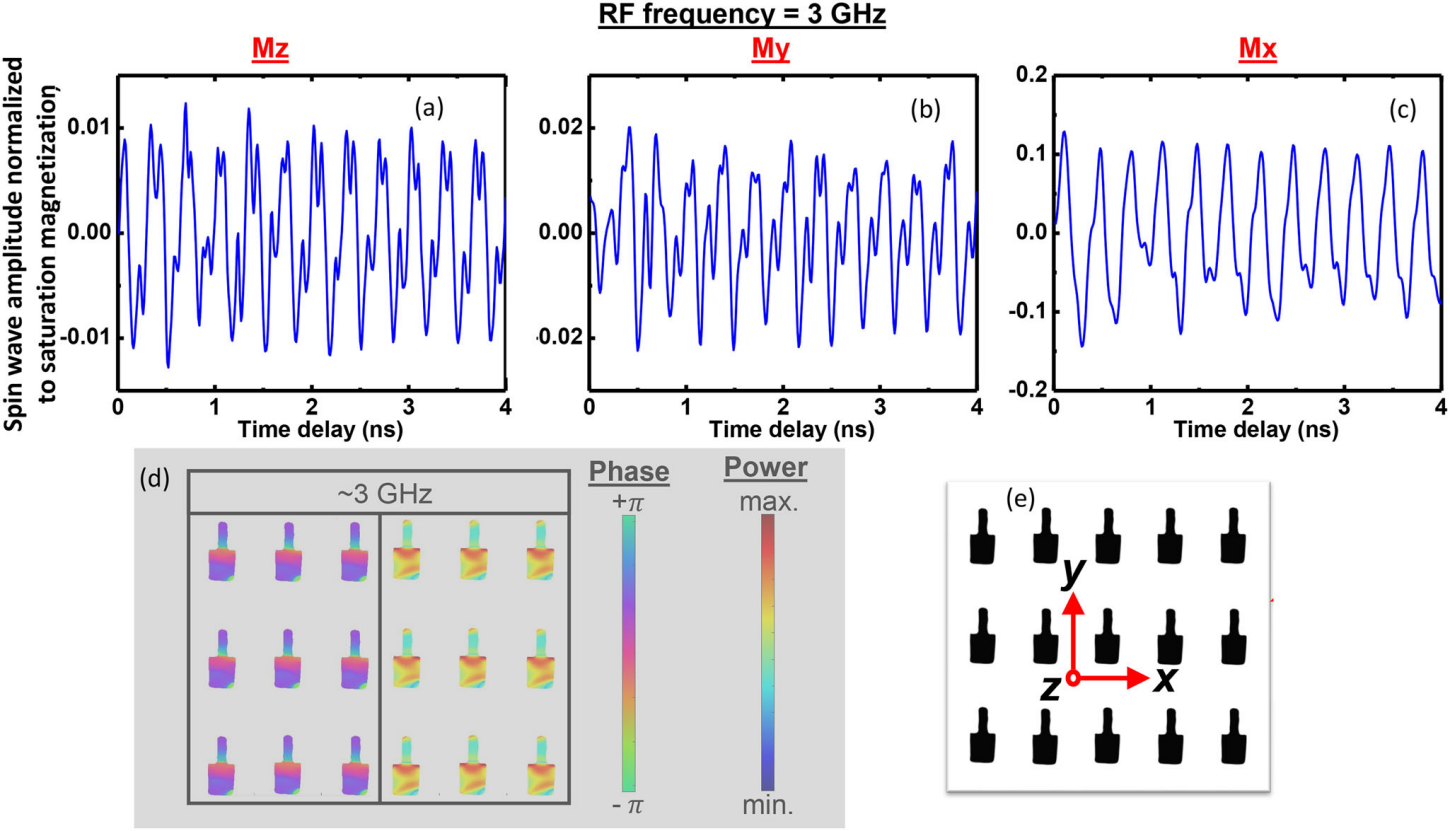
Simulated magnetization oscillations (spin waves) in a nanomagnet at an ac current frequency of 3 GHz. (a) z‐component (along the thickness); (b) y‐component (along the ledge); (c) x‐component (perpendicular to the ledge; (d) phase (left panel) and power (right panel) profiles of the spin waves inside a nanomagnet; (e) designation of the coordinate axes x, y and z.

The second feature to note is that the radiation pattern is different in the three different planes ‐ the plane of the nanomagnets and the two transverse planes. This feature also accrues from the anisotropy of the spin wave patterns excited in the nanomagnets by the spin Hall effect. To study this anisotropy, we used the micromagnetic simulator OOMMF package to find the magnetization components along the three coordinate axes $M_{x}\left(t\right)$, $M_{y}\left(t\right)$ and $M_{z}\left(t\right)$ as a function of time for three different frequencies of the pumping current: 3, 4 and 6 GHz. These plots are shown in Figure 6 for 3 GHz. They are independent of the initial magnetization states within the nanomagnets. It is interesting to note that the oscillations (and hence the spin waves) have much larger amplitude along the x‐direction than along the other two transverse directions (y and z). This cannot be explained by the mere fact that the nanomagnets have in‐plane anisotropy and hence the in‐plane magnetization will be larger than the out‐of‐plane magnetization. If that were the case, then the x‐component should not have been five times larger than the y‐component, given that both x‐ and y‐directions are in‐plane directions. This unusual feature is observed at all three frequencies of 3, 4 and 6 GHz. Only the 3 GHz results are shown in Figure 6, while the other two can be found in Section S8. This feature is actually due to the *shape* anisotropy of the nanomagnets, which have ledges in the y‐direction and not in the x‐direction. Hence the spin wave amplitude is larger in the x‐direction than in the y‐direction, even though both are in‐plane directions.

Because the spin waves have different amplitudes in different directions, the nanomagnetic radiator acquires “internal anisotropy” which makes the radiation pattern different in different planes, and also anisotropic in each of these planes. This raises an intriguing possibility. If we could change the direction of the spin–orbit torque on the fly, we might be able to *steer the radiated beam*, thereby mimicking an actively electronically scanned array (AESA radar). That is not possible in this configuration, but we have achieved this feat in another experiment by replacing the heavy metal with a topological insulator [41].

In Figure 6, we also show the power and phase profiles of the spin waves excited in the nanomagnet at 3 GHz excitation, calculated by the procedure described in [42]. Surprisingly, these profiles are quite frequency‐dependent. For example, at 3 GHz, the spin wave power is concentrated at the edge of the nanomagnet abutting the ledge, while at 4 and 6 GHz (see Supporting Information) it is concentrated in the ledge and the edge facing away from the ledge. These features are responsible for the *frequency dependence* of the radiation pattern (because the spin wave patterns are frequency‐dependent).

It would have been very desirable to show that the power radiated by the nanomagnets correlates with the power in the spin waves, but this is very difficult to establish. We found that the spin wave amplitude measured by TR‐MOKE increases with increasing input power provided to the antenna in the form of the ac current pumped into the Pt nanostrips. However, we cannot show convincingly that the power radiated by the nanomagnets increases with increasing input power similarly. The reason that we cannot show the latter convincingly is that when averaged over all directions, the difference in the power radiated by the real sample and the control sample is small. That difference is the power radiated by the nanomagnets, and to show that it is correlated with the input power (quantitative correlation) will require a degree of measurement accuracy that is not achievable. We know that the power received by the horn antenna placed at an arbitrary location increases if we increase the input power, but this merely provides a qualitative indication that the radiated electromagnetic power increases with the spin wave amplitude. Unfortunately, it does not provide a quantitative relationship. This is something beyond our current capability.

In future, we will attempt to extract the quantitative relationship, perhaps with different sets of measurements, but at present, we can only claim a qualitative relationship, not a quantitative one, because of our measurement limitations.

## 3 Receiving Antenna

When electromagnetic (EM) radiation is incident on a ferromagnet, it excites spin waves in the latter. This can generally happen in two ways. First, the oscillating magnetic field in the EM radiation can excite spin waves [43, 44, 45]. Second, parametric pumping can excite spin waves at half the frequency of the oscillating magnetic field [45, 46, 47]. An additional consideration comes into play when the ferromagnet is patterned into *nanostructures* and arranged in a periodic 2D array (sometimes referred to as a “magnonic crystal”). In this case, very specific confined modes (such as center modes, edge modes, quantized modes, etc.) can be excited in the nanomagnets by the EM fields. They are of two types – *intrinsic* and *extrinsic* [48]. The intrinsic mode frequencies are determined by such parameters as the shape, size and material composition of the nanomagnets [49], as well as the pitch of the array, and may not have any relation to the incident EM wave frequency. The extrinsic mode frequency, however, is the same as that of the excitation, namely the incident EM wave and is determined only by the EM wave. Both intrinsic and extrinsic modes are spawned by transferring energy from the EM wave to the spin waves by photon–magnon coupling, which has recently been shown to be quite strong in these systems [34].

If the nanomagnets are in physical contact with a heavy metal that exhibits the spin Hall effect (e.g. Pt), then the EM‐excited intrinsic and extrinsic spin waves can *pump* spin into the heavy metal [50] at their own frequencies and that can cause a polychromatic ac voltage to appear across the heavy metal via the ac inverse spin Hall effect [50, 51, 52, 53, 54]. This voltage's frequency components will correspond to frequencies of the intrinsic and extrinsic modes that are excited in the nanomagnets by the incident EM field. The appearance of this voltage signals the presence of the EM radiation and hence implements a receiving antenna.

**FIGURE 7:**
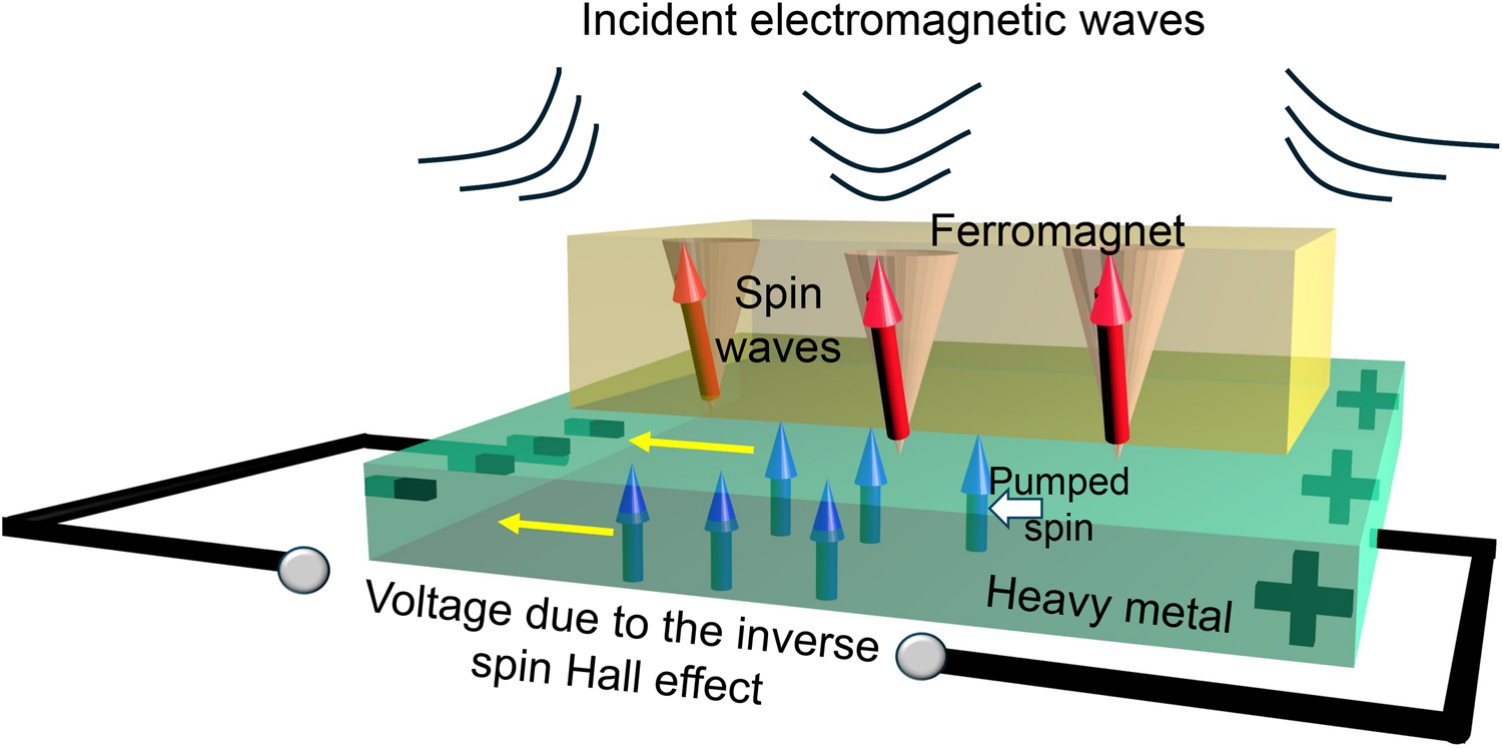
Operating principle of the receiving antenna based on ac spin pumping and the ac inverse spin Hall effect. EM radiation excites spin waves in the ferromagnet, which pumps spin into the heavy metal layer and that causes an ac voltage to appear across the latter, which can be electrically detected to implement a receiving antenna.

The excitation and amplification of intrinsic spin wave modes and generation of an extrinsic mode in a magnonic crystal by a surface acoustic wave (SAW) was recently demonstrated by us [48]. In a magnetostrictive nanomagnet, the SAW effectively produces an alternating magnetic field directed in the direction of SAW propagation [48]. The EM wave also generates an alternating magnetic field in the nanomagnet and therefore is similar in effect to a SAW. Consequently, it will also excite and/or amplify intrinsic modes and produce an extrinsic mode at the EM wave frequency in the nanomagnets. Both or either of the effects can be manifested. These spin wave modes will pump spin into the heavy metal nanostrips that are in contact with the nanomagnets and cause an ac voltage to appear across the heavy metal owing to the inverse ac spin Hall effect. That realizes receiving antenna operation. The generated voltage will contain frequency components corresponding to the frequencies of the excited intrinsic and extrinsic spin wave modes due to the EM wave. The underlying principle of this voltage generation (i.e. the receiver antenna operation) is illustrated in Figure 7.

### 3.1 Testing the Receiver Function

As always, we fabricated two samples – one with the nanomagnets and the other without. We call the former the “real sample” and the latter the “control sample”. We compare the signals received from the two samples to eliminate spurious effects. If our theory is correct, the control sample will not generate any ac voltage output, but the real sample will.

**FIGURE 8:**
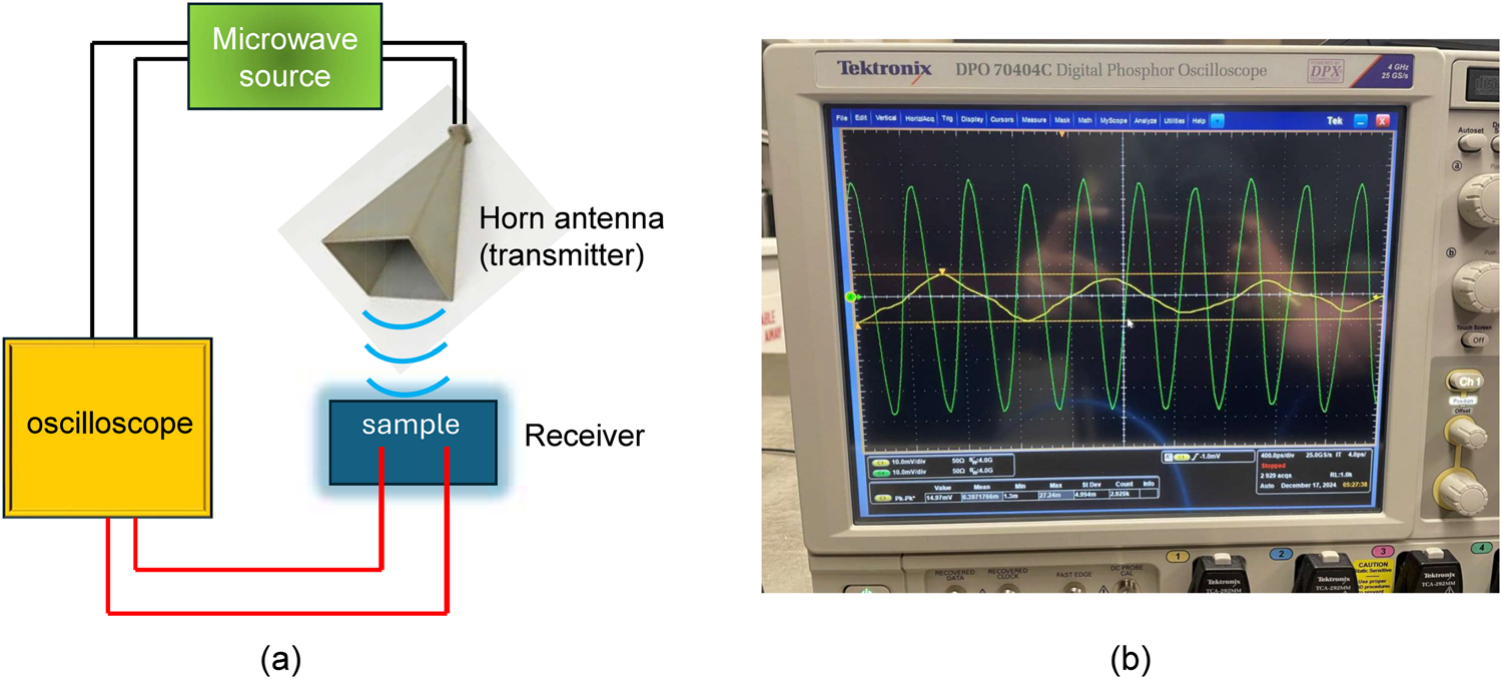
(a) Schematic of the experimental set up. (b) Oscilloscope traces for the real sample when the separation between the horn antenna and the sample is 6 in. The green trace is the signal fed to the horn antenna (transmitted signal) and the yellow trace is the output measured between the two contact pads of the real sample (received signal). They are both on the same scale of 10 mV/div. The waveforms and periods are very different since the output has intrinsic and extrinsic modes mixed into it. In this case, the two signals are almost out of phase with each other, and the time difference between a peak of the yellow trace and the closest peak of the green trace is 0.13 ns. The ratio of the peak‐to‐peak amplitudes of the two signals $V_{in}/V_{out}$ is ∼4:1.

**FIGURE 9:**
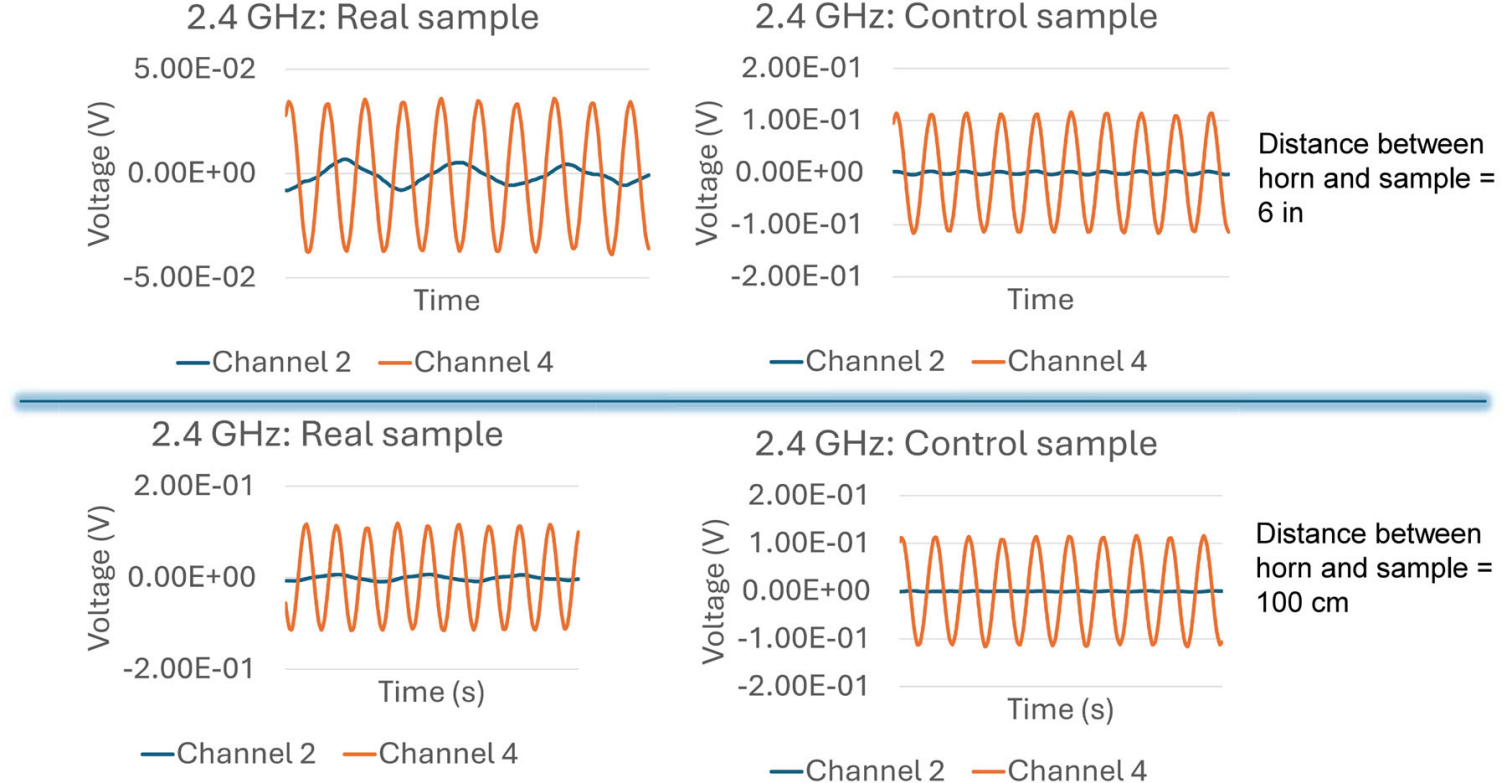
Digitized oscilloscope traces of the input signals fed to the horn antenna (in orange) and the output signals produced at the two output contact pads (in blue). The upper panel corresponds to a horn‐sample separation of 6 in and the lower panel to 100 cm. The input signal is fed at channel 2 of the oscilloscope and the output signal at channel 4. The left panel corresponds to the real sample and the right panel to the control sample.

Both samples were illuminated with a 2.4 GHz and a 1.5 GHz microwave signal transmitted with a horn antenna fed from a microwave source emitting 5 dbm of power as shown in Figure 8a. The two output pads of the sample and the port of the microwave source were connected to two different channels of a microwave frequency digital oscilloscope. The first channel displays the waveform of the incident signal emanating from the horn antenna and the second channel displays the waveform of the received signal measured between the two contact pads. We observed the oscilloscope traces in the two channels for two different separations between the sample and the transmitting horn antenna – of 6 inches and 100 cm. The digital outputs of the oscilloscopes are plotted in Figure 9 for the two separations at 2.4 GHz excitation. The results for both the real sample and the control sample are shown. We chose the frequency 2.4 GHz since it conforms to = standard Bluetooth and Wi‐Fi.

### 3.2 Discussion of the Receiver Function

Several features are observed in Figure 9. Looking at the *control* sample results, we find that a small ac voltage appears between the output terminals when the distance between the horn and the sample is 6 inches. Nothing detectable appears when the distance increases to 100 cm (at the oscilloscope scale of 10 mV/div). More importantly, at 6 inches separation, the ac voltage detected at the control sample's output has the *same waveform* as the EM signal radiated by the horn antenna, and there is virtually *no phase shift* between them. Therefore, this received signal in the control sample is most likely due to direct *electromagnetic pickup* through the air that has nothing to do with the receiver functionality. Signal from the horn antenna is traveling directly through air to the output contact pads of the control sample.

The story with the *real* sample is very different. There are many differences between the signal fed to the horn antenna (transmitted signal) and the signal appearing between the output contact pads (received signal): (1) they do not have the same frequency, (2) they do not have the same waveform and (3) there is a clear phase shift between the two. This eliminates electromagnetic pickup as the source of the output signal. It is the signal produced by transduction of EM waves to spin waves, followed by spin pumping, followed by the ac inverse spin Hall effect that causes the ouput voltage. The existence of this signal establishes the detector (or receiving antenna) functionality.

#### 3.2.1 Frequency Components of the Received Signal

**FIGURE 10:**
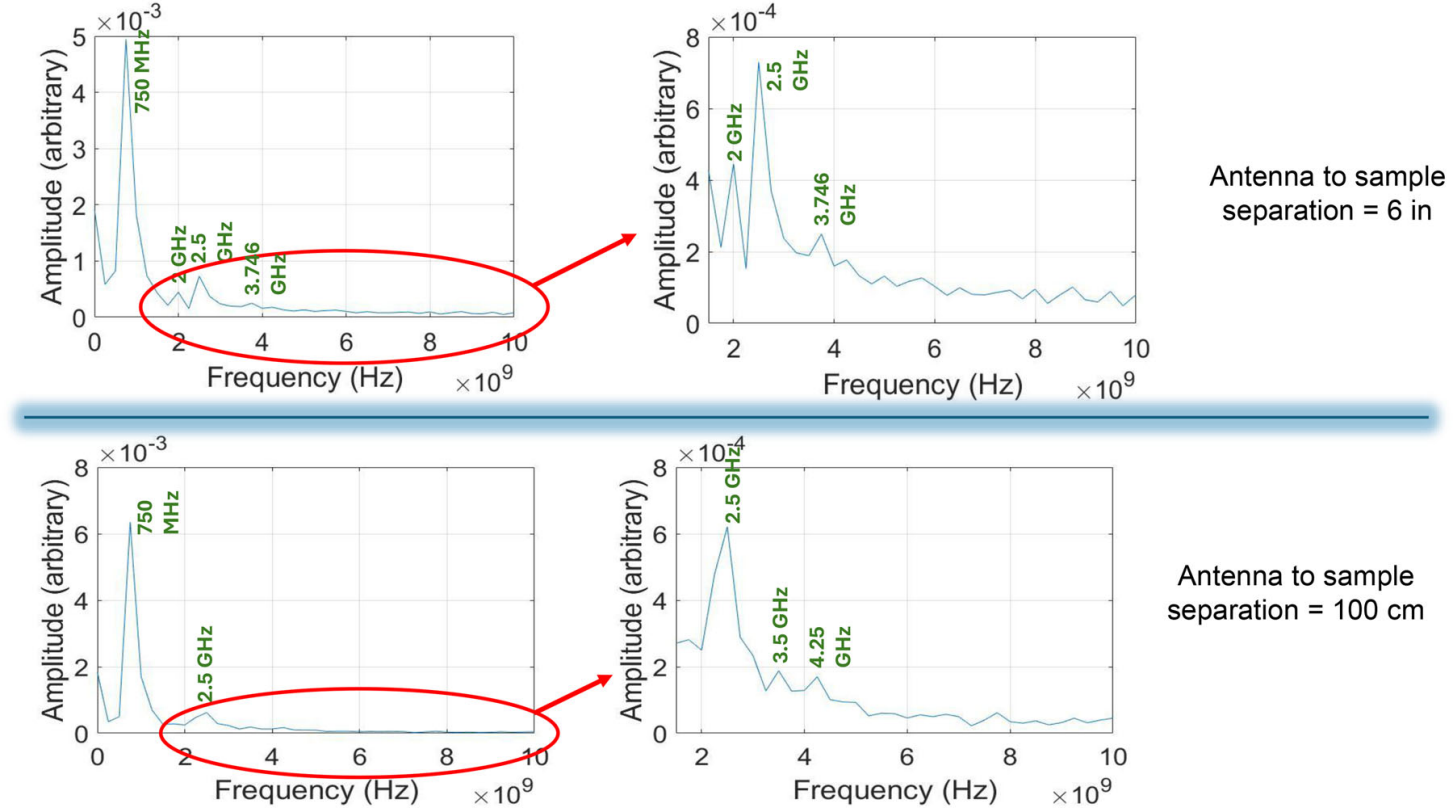
Fast Fourier transform (FFT) of the received signal when the EM excitation frequency is 2.4 GHz. The upper panel shows the result when the horn‐to‐sample separation is 6 in and the lower panel shows the result when the separation is 100 cm. The left figure in either panel is the FFT showing all the peaks, whereas the right figure is a plot of the satellite peaks where the main peak at 750 MHz has been intentionally suppressed.

In Figure 10, we show the fast Fourier transform of the received signal in the real sample at 2.4 GHz excitation for the case when the horn‐sample separation is 6 in and also for the case when the separation is 100 cm. Note that the dominant peak is at 750 MHz, which is *not* the EM signal frequency of 2.4 GHz. There is also a satellite peak at 2.5 GHz (close to the 2.4 GHz frequency of the incident radiation). Both peaks show up independent of the horn‐to‐antenna separation, i.e., both at 6 in and 100 cm separation. There are other smaller peaks whose frequencies are not separation independent.

The 750 MHz peak also shows up when the excitation frequency is changed to 1.5 from 2.4 GHz (see the Supporting Information). We believe that it is an *intrinsic* spin wave mode in the nanomagnet array, which is excited by the EM wave. Its frequency is determined by the size, shape and material composition of the nanomagnets [48, 49] as well as other array parameters, but is independent of extrinsic parameters such as the EM frequency or separation between the transmitter and the receiver. It is excited by energy transfer from photons to magnons [34].

#### 3.2.2 Covert Communication

The Fourier transforms of the received signal at either 2.4 GHz incident radiation or 1.5 GHz incident radiation do have frequency components at ∼2.4 and 1.5 GHz, respectively. Thus, an intended receiver, who is aware of the transmission frequency can always use a narrow band‐pass filter centered at the transmission frequency to filter out the transmitted signal from the received signal. An unintended receiver (eavesdropper), on the other hand, will have no knowledge of the transmission frequency and hence cannot employ this strategy to filter out and receive the transmission. They will be confounded by the additional frequency components. This introduces an element of “stealth”, whereby a message can be transmitted over a public channel and yet concealed from an eavesdropper. Covert communication using polarization states of similar antennas have been demonstrated by us in the past [38] and this is another variant.

We also note that the receiver can be utilized for other purposes as well, such an energy harvesting and wireless power transfer [39]. In this case, the polychromatic received signal poses no issues.

Curiously, there are some modes at 2.0, 3.5, 3.75 and 4.25 GHz which depend on the separation between the transmitter and the receiver. These cannot be intrinsic modes because they are not independent of separation. They could be due to *vortex modes* caused by strain pulses generated in the magnetostrictive nanomagnets by the EM radiation. The EM radiation very likely causes some periodic heating and cooling of the nanomagnets, which, in turn, can cause periodic strain in the nanomagnets due to the difference in the thermal expansion coefficients of the nanomagnet material and the substrate. It has been shown that this can spawn vortex modes in magnetostrictive nanomagnets [40]. If we change the separation between the transmitter and receiver, the heating rate will change, and that will change the vortex modes and their frequencies. We emphasize that this is somewhat speculative but can explain the observed separation dependence.

Looking at the results for the control sample in the right panel of Figure 9, we do not see any measurable signal produced between the contact pads at the horn‐sample separation of 100 cm (when both oscilloscope channels have the same amplification of 10 mV/div) and the signal at the separation of 6 in clearly has only one frequency component (or at least a dominant frequency component) at 2.4 GHz, which is the signal frequency. There is no frequency component at 750 MHz or 2.5 GHz since the control does not have any nanomagnet. The signal produced between the pads in the control sample is almost surely due to electromagnetic pick up because it is in phase with the input signal (within our measurement tolerance) and has the same frequency.

#### 3.2.3 Receiver Gain

We can calculate the receiver gain $G_{r}$ at 2.4 GHz incident frequency from the formula [55]

(1) $$\frac{P_{r}}{P_{t}}\approx \frac{V_{out}^{2}}{V_{in}^{2}}=G_{t}G_{r}\frac{\lambda^{2}}{4\pi R^{2}}$$

where λ is the wavelength, R is the separation between the antenna and the sample, $P_{r}$ and $P_{t}$ are the received and transmitted power, respectively, $V_{out}$ and $V_{in}$ are the amplitudes of the waveform in the two oscilloscope channels, and $G_{t}$ is the gain of the transmitting ETS 3115 horn antenna at 2.4 GHz, which is 9.6 dB = 9.12, as provided by the manufacturer. This yields

$$G_{r}=0.1277=-8.9db.$$

We can check if we get a very different gain from the 100 cm separation data. In this case $Vin/V_{out}\approx 25$. Hence, the gain is

$$G_{r}=0.116=-9.3db,$$

which is very close to the previous value, showing that the gain is roughly separation‐independent at 2.4 GHz incident frequency (for these separations), and is approximately –9 db.

There are theoretical limits on the transmitting gains of conventional antennas that radiate via classical fluctuating electrical dipoles. Although they vary slightly depending on the exact type of the antenna, it is generally of the order [21, 22]

(2) $$G_{t}^{max}=A/{\left(2\pi \lambda \right)}^{2}+\sqrt{A}/\left(\pi \lambda \right)$$

where A is the antenna area, and λ is the free space radiated wavelength. Because of the principle of reciprocity, we would expect the same limit to apply to the receiving gain. In our case, this limit turns out to be about 3.22$\times 10^{-5}$ which is ∼ –45 db. Our gain is 36 db larger, i.e., about *4000 times larger* than the theoretical limit for conventional antennas. However, this is not surprising for unconventional antennas that operate by unconventional means because in the past they have shown gains that exceed the theoretical limit in Equation (2) by more than two orders of magnitude [26].

## 4 Conclusion

We have demonstrated a spin Hall nano‐antenna (SHNA) which is an analogue of the celebrated spin Hall nano‐oscillator (SHNO) – the difference being that the latter delivers an alternating signal to a load, whereas the former radiates an alternating signal into the surrounding medium. The transmitting SHNA is actuated by periodic spin–orbit torque (SOT) generated in nanomagnets by injecting an alternating current into a heavy metal nanostrip in physical contact with the nanomagnets. The alternating SOT, arising from the alternating spin Hall effect in the heavy metal, generates confined spin waves (magnons) in the nanomagnets that radiate electromagnetic waves (photons) in space via magnon–photon coupling. We have provided a classical phenomenological model for this effect in Section S12. We have also confirmed that the SHNA effect is fully repeatable by demonstrating the phenomenon in two different samples fabricated at two different times (see Supporting Information).

We understand that even though the SHNA “effect” itself is fully reproducible and consistent, the “details” (radiation pattern, etc.) are not and vary from sample to sample. Why that happens is explained in Section S15. This is to be completely expected since the details depend on the precise size, shape and arrangement of the nanomagnets, which are impossible to reproduce exactly across different devices with the current state of fabrication technology. As this technology matures, we expect the device‐to‐device variations to decrease. Here, we are interested in the “physics” of this new phenomenon and not the “engineering” of the details, which is premature at this nascent stage.

The SHNA can be more than an order of magnitude smaller than the radiated electromagnetic wavelength and yet emit efficiently because of the unconventional antenna principle it employs. Hence, it is ideal for embedded applications where the antenna has to be much smaller than the wavelength (medically implanted devices, wearable electronics, stealth devices, etc.) and yet must radiate with acceptable efficiency. We have also shown that while these antennas can be viewed as “point sources” because they are much smaller than the electromagnetic wavelength, they nonetheless radiate anisotropically because the spin waves that are excited in the nanomagnets are anisotropic in nature.

Finally, we have demonstrated both a transmitting antenna and a receiving antenna by exploiting *reciprocal effects* (spin–orbit torque vs. spin pumping, spin Hall effect vs. inverse spin Hall effect). This duality completes a comprehensive and compact transceiver system.

## 5 Methods and Materials

### 5.1 Nanomagnet Array Fabrication

The lithium niobate substrate is first cleaned in acetone and isopropyl alcohol and spin‐coated (spinning rate ≈2500 rpm) with a single layer polymethyl methacrylate (PMMA) resist and subsequently baked at 110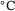 for 2 min. Next, electron beam lithography is performed using a Raith Voyager Electron Beam Lithography system having an accelerating voltage of 50 kV and beam current of 300 pA to open windows for deposition of the Pt nanostrips. The resists are subsequently developed in methyl isobutyl ketone and isopropyl alcohol (MIBK‐IPA, 1: 3) for 60 s, followed by a cold isopropyl alcohol (IPA) rinse. A 5 nm‐thick Ti adhesion layer is deposited on the patterned substrate using electron beam evaporation base pressure 2.3$\times 10^{-7}$ Torr, followed by the sputtering of Pt at a base pressure of 10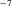 Torr. The lift‐off is carried out by remover PG solution (a proprietary solvent stripper).

Cobalt nanomagnets are fabricated similarly in a second layer of lithography and aligned with the Pt. nanostrips using previously delineated alignment marks.

### 5.2 Antenna Measurements

Measurements of antenna radiation patterns are carried out in an AMS‐8701 Anechoic Chamber, Antenna Measurement System, using a 3164–10 Open Boundary Quad‐ridged Horn Antenna. The sample (antenna) is always placed at a distance of 284.5 cm from the horn antenna, which ensures that we are measuring the far‐field radiation pattern at all frequencies.

## Author Contributions

R. F. fabricated the samples. R. F. and M. S. made the antenna measurements. P. K. P and A. K. M. carried out the TR‐MOKE measurements. S. B., E. T. and A. B. supervised the project and verified the data. S. B. conceived the idea. All authors contributed to writing the paper. R. F., M. S. and P. K. P. contributed equally.

## Funding

US National Science Foundation under grant ECCS‐2235789. Virginia Commonwealth University Commercialization Fund. Department of Science and Technology, Govt. of India (grant no. DST/NM/TUE/QM‐3/2019‐1C‐SNB). Indo‐US Science and Technology Fund Center grant “Center for Nanomagnetics for Energy‐Efficient Computing, Communications, and Data Storage” (IUSSTF/JC‐030/2018). The Council of Scientific and Industrial Research (CSIR), Govt. of India.

## Conflicts of Interest

The authors declare no conflicts of interest.

## Supporting information

Supporting file

## Data Availability

The data that support the findings of this study are available from the corresponding author upon reasonable request.
