# Supplementary material for: Spin Hall Nano‐Antenna
Source: Adv Sci (Weinh). 2026 Feb 25;13(23):e20505. doi: 10.1002/advs.202520505 (PMC13104134; doi:10.1002/advs.202520505)
Supplement: Supplementary file 1 — Supporting file [file ADVS-13-e20505-s001.pdf]

Supporting Information: Spin Hall Nano-Antenna

Raisa Fabiha[1], Pratap Kumar Pal[2], Michael Suche[1], Amrit Kumar Mondal[2], Erdem Topsakal[1], Anjan Barman[2] and Supriyo Bandyopadhyay[1]

[1]Department of Electrical and Computer Engineering, Virginia Commonwealth University, Richmond, VA 23284, USA

[2]Department of Condensed Matter and Materials Physics, S. N. Bose National Center for Basic Sciences, Kolkata 700116, INDIA

The information presented here are in support of the material presented in the main paper.

1. Time-resolved Magneto-Optical Kerr Effect Spectroscopy

The investigation of ultrafast magnetization dynamics of the samples is conducted by employing a custom-built TR-MOKE microscope based on a two-color collinear pump–probe technique under ambient conditions.

A small part of the fundamental laser beam (λ_{probe} = 800 nm, fluence = 2 mJ cm^{-2}, pulse width = 80 fs) from a mode-locked Ti-sapphire laser (Tsunami, Spectra-Physics) is used to probe the polar Kerr rotation (hereafter referred to as the probe beam) and its frequency-doubled counterpart (λ_{pump} = 400 nm, fluence = 16 mJ cm^{-2}, pulse width = 100 fs), referred to as the pump beam, is used to excite the magnetization dynamics of the samples. The Kerr rotation signal is measured using an optical bridge detector (OBD) as a function of the time delay between the pump and probe beams. Achieving spatial overlap of these two beams is critical. The slightly defocused pump beam (of diameter ~ 1 μm) at the focal plane of the tightly focused probe beam (of diameter ~ 800 nm), is made collinear and overlapping on the sample plane using a microscope objective of numerical aperture 0.65. The OBD isolates and simultaneously measures both the reflectivity and Kerr rotation in a phase sensitive manner with the help of two lock-in amplifiers to attain high sensitivity. To maintain temporal synchrony, the pump beam is subjected to periodic modulation at a frequency of about 2 kHz by a mechanical chopper. This modulation frequency serves as a reference frequency and is conveyed to the lock-in amplifiers, anchoring the phase relationships within the system. Further, the probe beam is systematically positioned at the precise center of the pump beam using an x–y–z piezoelectric scanning controller, guided by a feedback loop and a white-light illumination system, enhancing the fidelity of the experiment. The experimental time window of 2 ns was found to be sufficient to

resolve the spin wave peaks with a temporal resolution of 10 ps from the fast Fourier transform (FFT) of the bi-exponential background subtracted time-resolved traces.

2. Micromagnetic simulations

Micromagnetic simulations were carried out with the open source OOMMF software package. The discretized array featured rectangular parallelepiped-shaped cells (3 × 3 × 6 nm^3) with a two-dimensional (2D) periodic boundary condition. The unit cell length was kept below the exchange length of Co (~4.93 nm), to allow the incorporation of exchange interactions. Simulation parameters used included saturation magnetization, M_s = 1400 emu cc^{-1}, anisotropy constant, K = 0, gyromagnetic ratio, γ = 17.6 MHz Oe^{-1}, the exchange stiffness constant, $A_{ex} = 3.0 \times 10^{-6}$ erg cm^{-1}, and damping coefficient, α = 0.008.

3. Sample Fabrication

The samples are fabricated with multi-level e-beam lithography for patterning using a Raith e-beam writer, followed by e-beam evaporation of metals (Co, Pt) and finally lift-off. Fig. S1 is a scanning electron micrograph of the nanomagnets showing the overlaying of the Pt strip on the ledges very clearly.

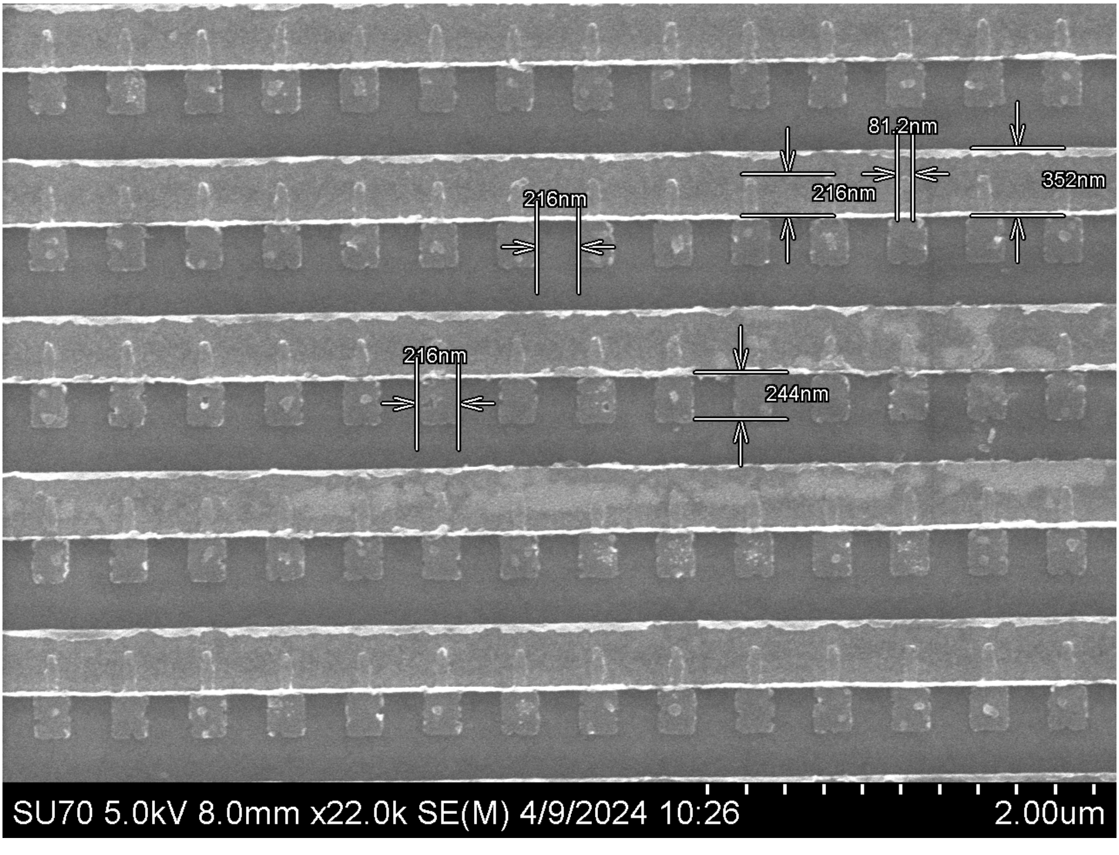

Fig. S1: Scanning electron micrograph of the samples showing the various dimensions.

4. Variability of high frequency Kerr oscillation spectra

In the TR-MOKE measurements, only two neighboring nanomagnets are sampled at a time because of the laser spot size and the Kerr oscillations are measured within these two nanomagnets. When a different nanomagnet pair is sampled, the mode at the frequency of the driving ac current remains unchanged in frequency, but the others (occurring at higher frequencies) experience red or blue shift. For example, at 3 GHz excitation frequency, the spin wave mode at 3 GHz is found in all sampled nanomagnet pairs (or all laser spot position) as shown by the dashed black line, but the higher order mode at ~4 GHz is found only in one position (Position 2) and not in other positions (or other nanomagnet pairs) [as shown by the thin red vertical line in Fig. S2]. The higher frequency modes are not excited by the ac current (or the associated periodic spin-orbit torque) but are probably vortex modes generated by strain pulses [1] which are produced in the magnetostrictive nanomagnets by the periodic heating and cooling by the pump and probe laser beam in the TR-MOKE setup. The frequencies of these modes depend on the diameter and thickness of the nanomagnet [1] and since these two parameters vary slightly from one nanomagnet to another, there is some variability in the frequencies of the higher order modes. As a result, when these higher frequency peaks are ensemble averaged over ~570,000 nanomagnets in a sample, *they wash out* and consequently we do not see their signatures in the electromagnetic radiation spectra. We only see the signature of the mode at the frequency of the ac current since its frequency does not vary from one nanomagnet to another and hence it survives ensemble averaging.

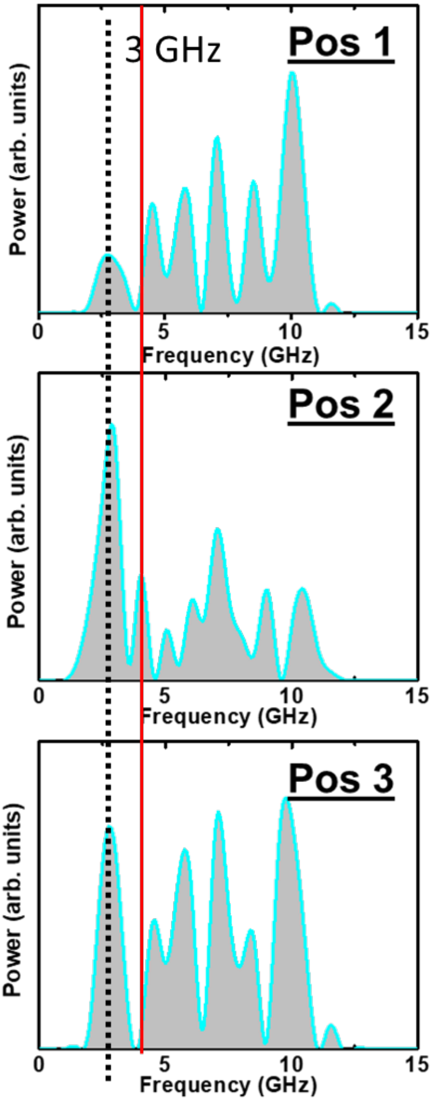

Fig. S2: Spin wave spectra measured with TR-MOKE in three different nanomagnet pairs.

5. Radiation pattern measurement setup

The radiation patterns were measured in an AMS-8701 Anechoic Chamber, Antenna Measurement System using a 3164-10 Open Boundary Quad-ridged Horn Antenna. The set up is shown in Fig. S3.

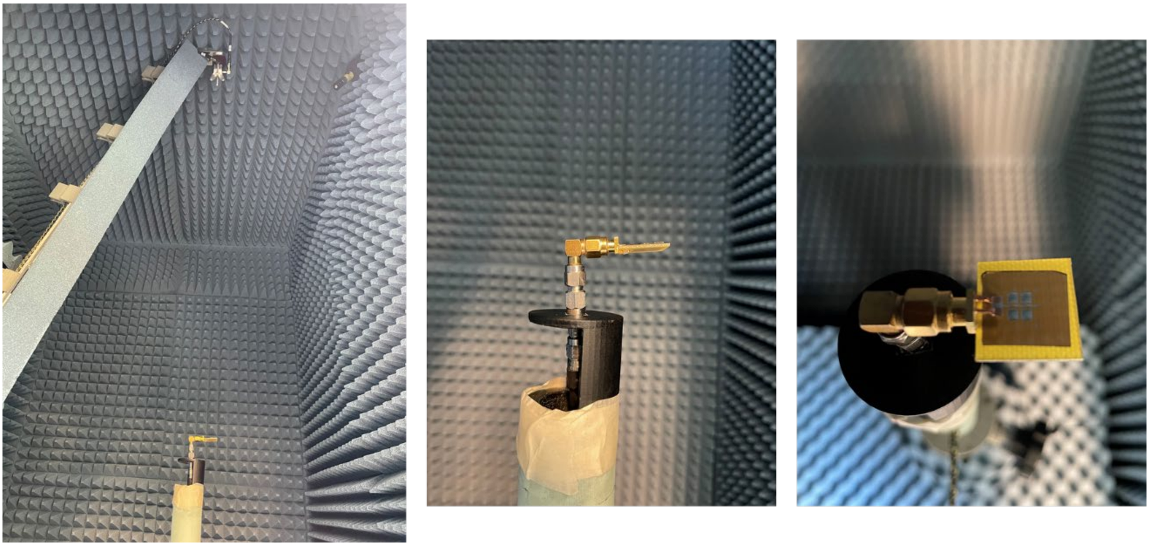

Fig. S3: Sample placement within the anechoic chamber.

6. Screenshots of the received electromagnetic spectra at the horn antenna detector from the real and control samples when the ac current frequency is 3 GHz

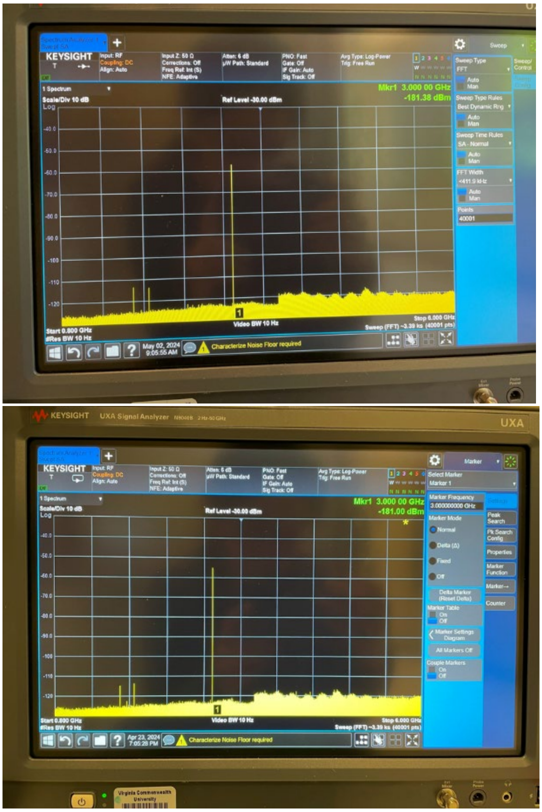

Fig. S4: Screenshots of the received radiation at the horn antenna taken from the spectrum analyzer. (Left) real sample and (right) control sample.

7. **Radiation patterns of the real and control samples at 3 GHz measured in the two planes that are transverse to the plane of the nanomagnets**

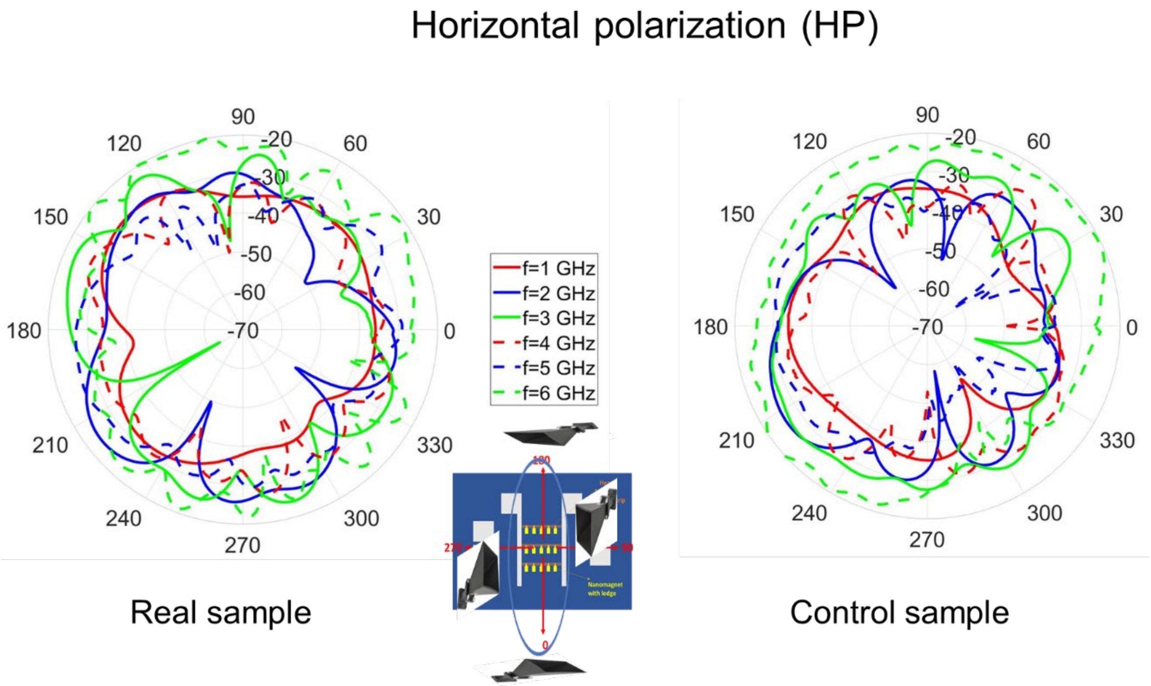

(a)

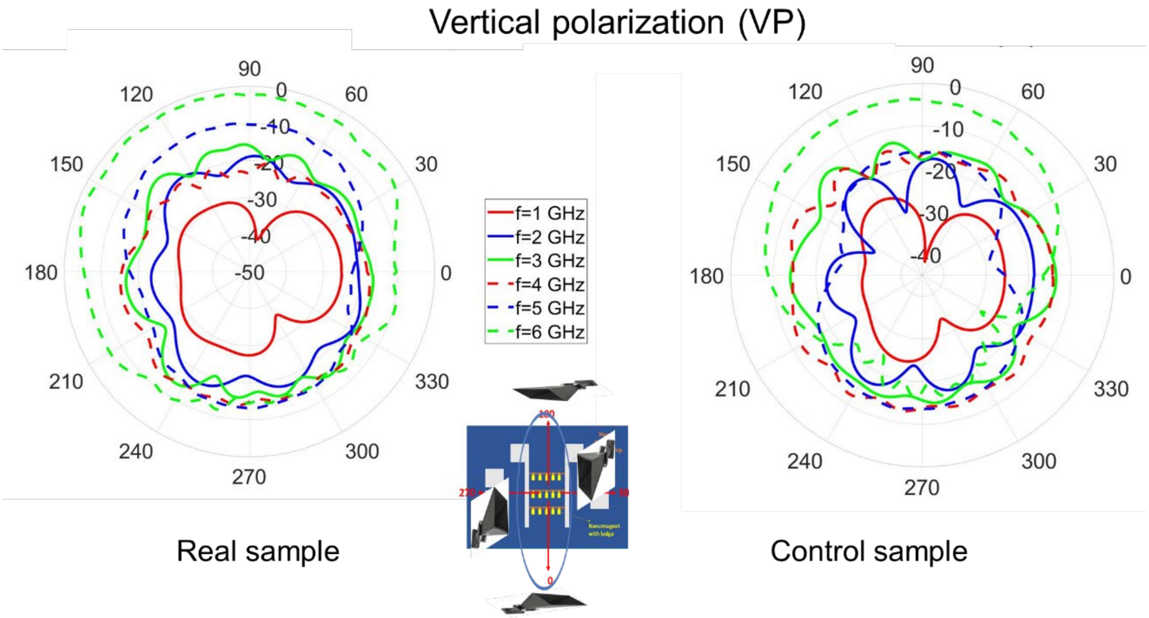

(b)

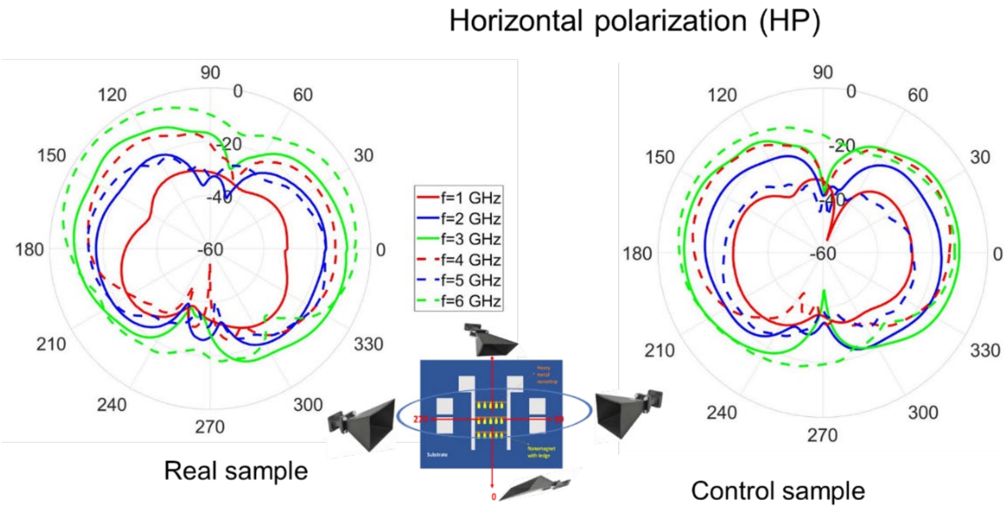

(c)

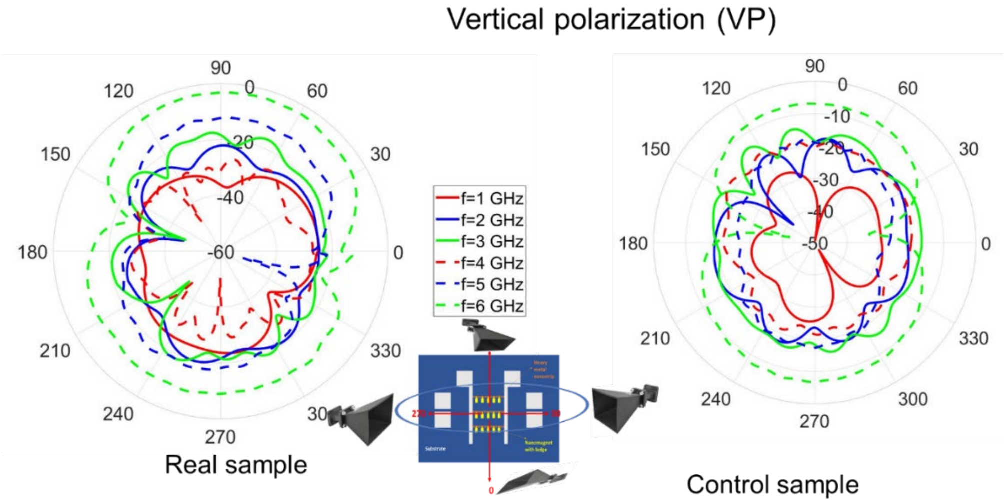

(d)

Fig. S5: Radiation patterns in the two planes perpendicular to the plane of the nanomagnets shown for both horizontal and vertical polarizations.

8. Magnetization oscillations and the power/phase profiles of the spin waves excited within a nanomagnet at 4 GHz and 6 GHz ac current frequency

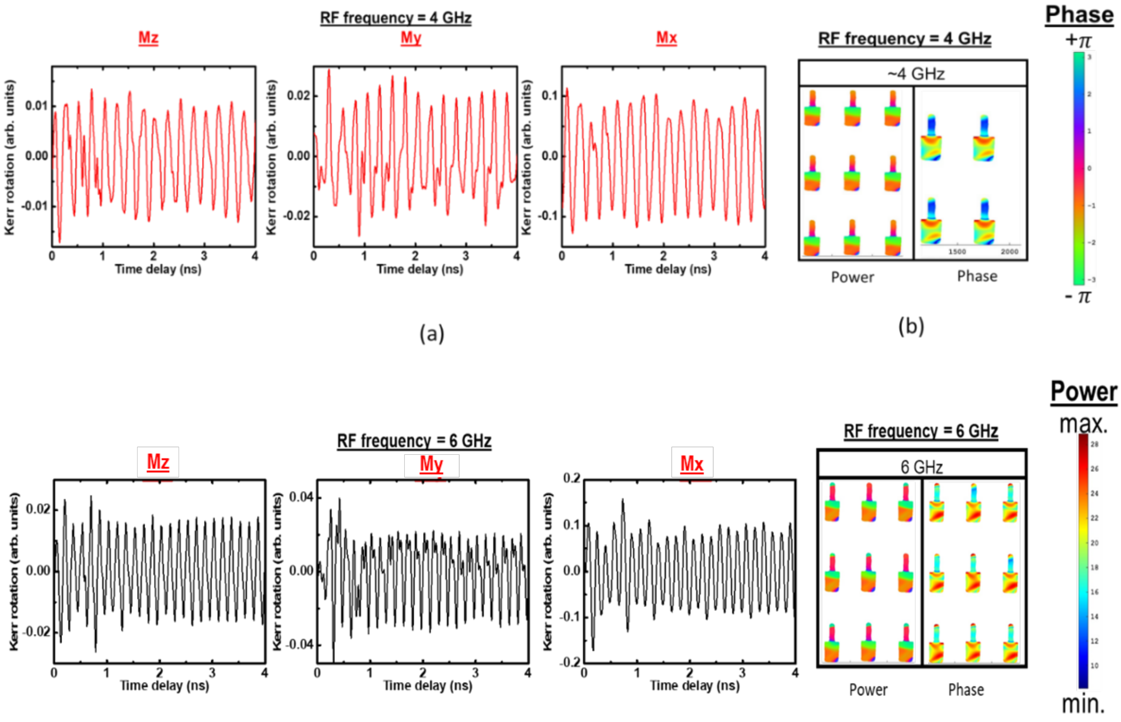

Fig. S6: (a) The oscillations of the three scalar components of the magnetization simulated from OOMMF and (b) the power/phase profiles of the associated spin waves in an array of 3 × 3 nanomagnets calculated with in-house software. The top panel shows the results for 4 GHz and the bottom panel for 6 GHz of ac current frequency (which is the frequency of the spin orbit torque). Note that the oscillation component in the direction perpendicular to the ledge (x-component) is always much larger than the other two components, making the spin waves anisotropic in nature. At both these two frequencies, the spin wave power is concentrated in the ledges and the edge of the nanomagnets facing away from the ledges.

9. Scattering parameter S_{11} spectrum

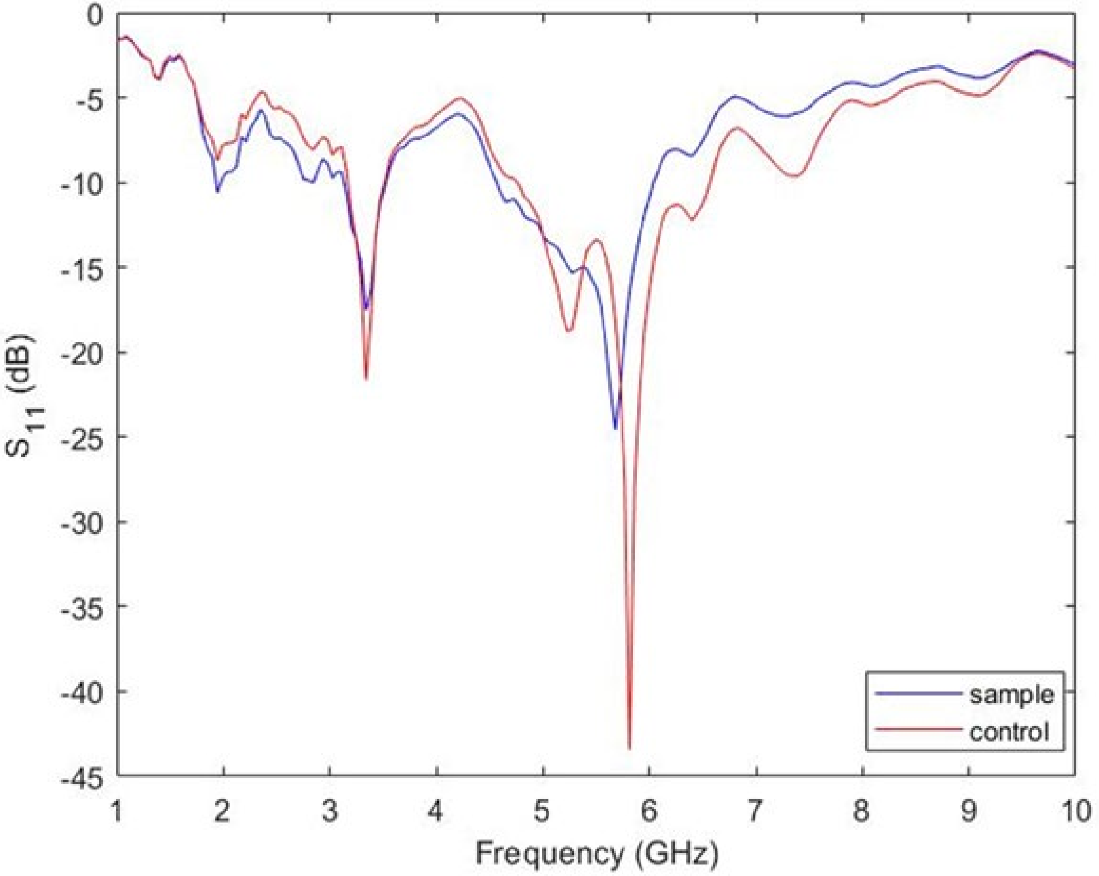

Fig. S7: Spectrum of the scattering parameter S_{11} of the sample measured with a vector network analyzer.

10. Is the radiation due to the sample acting merely as an SHNO which also has spin waves that can radiate electromagnetic waves, or is it a different phenomenon?

The SHNO works very differently from the SHNA. In the SHNO, one uses a magnetic field (or something emulating a magnetic field) to cause precessional oscillations (spin waves) and these oscillations are sustained by a dc spin orbit torque which counteracts the material damping. *The frequency of the oscillations in a SHNO is determined by the magnetic field and not by any input signal*. Hence, even if the SHNO radiates electromagnetic waves, it can only radiate at **one frequency** which is the precessional frequency determined by the magnetic field. That frequency has no relation with the frequency of any input signal. An antenna, on the other hand, will always radiate at the frequency of the input signal. While the SHNO can radiate at only one frequency, the SHNA can radiate at many frequencies depending on the frequencies of the input signal.

Let us say that the fixed SHNO frequency is f_0 set by the magnetic field. If the SHNA is also radiating at f_0, then one might worry about SHNO artefact, but if the SHNA is radiating at

multitudes of frequencies $f_1, f_2, \ldots f_n$, which are different from f_0, then there can be no effect of SHNO which can radiate only at f_0. Since our antenna radiates at multiple frequencies – always the frequencies of the input signal – there is no possibility of any SHNO artefact.

11. Increase in the radiation efficiency of this antenna beyond the Harrington limit

Any conventional antenna will be subjected to the Harrington limit which would limit the radiation efficiency to roughly A/λ^2 where A is the emitting area and λ is the radiated wavelength. In our case, assuming optimistically that every nanomagnet radiates at 3 GHz, this quantity A/λ^2 is $(160\ \mu\text{m}/10\ \text{cm})^2 = 2.5\times10^{-6}$, which means that this antenna, had it been conventional, would have had a maximum efficiency of 2.5×10^{-6}.

We can make an order estimate of the radiation efficiency of the SHNA. The power received at the receiving antenna from the SHNA is (conservatively) of the order of 1 nW. The scattering parameters S_{11} at 3 GHz is -17 db = 0.02. The power from the source is 31 mW and the power coupled into the sample is $31\times(1-S_{11}) = 30.4$ mW. This is the input power.

The power emitted by the SHNA (P_t) is found from the relation [1]

$$P_r = P_t G_t G_r \left(\frac{\lambda^2}{4\pi R^2} \right), \tag{1}$$

where P_r is the power received at the receiving antenna (~ 1 nW), G_t is the gain of the transmitting antenna which is ~ -10 dbi (from Fig. 5) = 0.1, G_r is the gain of the receiving antenna which is known to be about 7 db (=5) at 3 GHz, λ is the wavelength at 3 GHz (= 10 cm) and R is the separation between the SHNA and the receiving antenna which is 284.5 cm. This yields that the power emitted by the antenna P_t is 255 μW. The intrinsic radiation efficiency is the ratio of the power emitted to the power coupled into the sample from the input, which is $255\ \mu\text{W}/30.4\ \text{mW} = 8.4\times10^{-3}$. *This is three orders of magnitude higher than the Harrington limit.*

The estimated intrinsic radiation efficiency of $\mathbf{8.4\times10^{-3}}$ is also an indicator of the strength of enhanced magnon-photon coupling, enhanced by relativistic interfacial spin orbit torque at the interface of the heavy metal and the nanomagnets, first reported in ref. [4] of the main paper. It is strong enough to beat the Harrington limit overwhelmingly.

There is also a Harington limit on the antenna gain given by $G_{\text{limit}} = A/(2\pi\lambda^2) + \sqrt{A}/(\pi\lambda)$. In our case, that would have been $\sim 5\times10^{-4} = -33$ db. Our maximum measured gain approaches -15 db, which beats the Harrington limit by 18 db or 63 times.

A number of other unconventional antennas that beat the Harrington limit have appeared in the literature. The nearest one is one demonstrated by us that is based on tripartite phonon-magnon-photon coupling [2]. The following table provides a comparison in the transmission mode.

Table: Comparison of the transmitting spin Hall nano-antenna with transmitting nano-antenna based on tripartite phonon-magnon-photon coupling

	**Max ratio of measured radiation efficiency to the Harrington limit**	**Max ratio of measured antenna gain to the Harrington limit**
**Tripartite nano-antenna**	$\sim 10^5$	˜650
**Spin Hall nano-antenna**	$\sim 10^3$	~60

12. Classical phenomenological theory of the SHNA

We follow refs. [3] and [4] to provide a classical phenomenological theory for the SHNA operation. This theory does not capture the quantum-mechanical nuances of magnon-photon coupling that causes the generated spin waves to emit electromagnetic waves, but provides a classical picture of how spin waves caused by spin-orbit torque [2] produces electromagnetic radiation.

Ref. [3] showed that alternating spin-orbit torque (SOT) due to the ac spin Hall effect in the Pt nanostrip will produce an effective time-varying magnetic field in the nanomagnets given by

$$\vec{H}_{eff}^{SOT}\left(\vec{r},t\right)=\frac{J\left(t\right)\hbar}{2e\mu_0 M_s d}\left(\underbrace{\eta_D \hat{m}\left(\vec{r},t\right)\times\hat{n}}_{\vec{H}_{damping}}+\underbrace{\eta_F\hat{n}}_{\vec{H}_{field-like}}\right), \qquad (2)$$

where $J(t)$ is the alternating current density through the Pt strip causing the ac-SOT, M_s is the saturation magnetization of the nanomagnet material, d is the thickness of the Pt strip, η_D and η_F are the empirical efficiencies of the damping- and field-like components of the spin-orbit torque, $\hat{m}\left(\vec{r},t\right)$ is the unit vector in the direction of magnetization and $\hat{n}$ is the unit normal to the interface between the Pt strip and the nanomagnet ledge. Note that the effective magnetic field has a damping component which is in the plane of the nanomagnet and a field-like component which is out-of-plane.

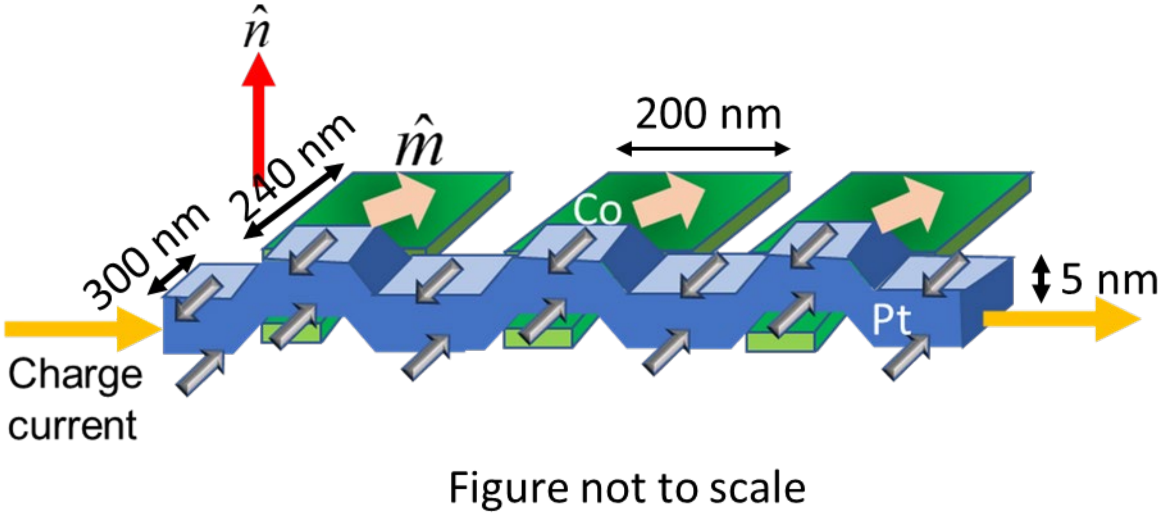

The Landau-Lifshitz-Gilbert (LLG) equation yields the time evolution of $\hat{m}(\vec{r},t)$ as

$$\frac{\partial \hat{m}(\vec{r},t)}{\partial t} = -\frac{\gamma}{1+\alpha^2}\left(\hat{m}(\vec{r},t)\times\vec{H}_{eff}(\vec{r},t)\right) - \frac{\gamma\alpha}{1+\alpha^2}\left[\hat{m}(\vec{r},t)\times\left(\hat{m}(\vec{r},t)\times\vec{H}_{eff}(\vec{r},t)\right)\right], \quad (3)$$

where γ is the gyromagnetic constant [2.21×10^5 (rad-m)(A-s)$^{-1}$], α is the Gilbert damping factor and $\vec{H}_{eff}(\vec{r},t)$ is the effective magnetic field in the nanomagnet due to SOT, shape anisotropy, dipole coupling between neighbors, exchange field and the thermal noise field. We neglect the thermal noise field since it is random and its effect will therefore be also random.

There is no magneto-crystalline anisotropy since the nanomagnets are amorphous and hence no contribution to $\vec{H}_{eff}(\vec{r},t)$ from magneto-crystalline anisotropy. Ref. [12] in the text considered an additional "induction" field contribution which we can neglect since the bulk of the nanomagnets lies outside the Pt strips and hence the effect of the induction field will be small. The dipole coupling field will be small since the nanomagnets are far apart (center to center distance ~0.55 μm) and the exchange field is also small since it is proportional to the number of spins (and hence the volume of the nanomagnet) which is small. In fact, because of the small size, the nanomagnets can be monodomain or contain few domains which will make the exchange field small. That leaves the shape-anisotropy field. Hence $\vec{H}_{eff}(\vec{r},t) = \vec{H}_{SOT}(\vec{r},t) + \vec{H}_{shape}(\vec{r},t)$.

Unfortunately, there is no analytical formula to calculate the shape anisotropy field in these odd shaped nanomagnets with ledges but we know that it is strong from Fig. 6 of the main manuscript since we find that the amplitude of the *x*-component of the magnetization is an order of magnitude larger than that of the *z*-component and 5 times larger than that of the *y*-component. That can only happen if the shape-anisotropy field is very strong and directed in the *x*-direction (or close to it). If the shape anisotropy field is very strong, then the instantaneous magnetization vector will always

tend to align along the instantaneous shape anisotropy field, making $\hat{m}(\vec{r},t)\times\vec{H}_{shape}(\vec{r},t)\approx 0$, where $\vec{H}_{shape}(\vec{r},t)$ is the shape-anisotropy field.

For the rest of the derivation, we follow ref. [4].

Solution of the LLG equation (3) yields the time-varying magnetic induction due to SOT given by $\vec{B}_{SOT}(\vec{r},t)=\mu_0 M_s \hat{m}(\vec{r},t)$. Here, we have assumed that the magnetic flux density is linearly proportional to the field, which is reasonable for order estimations. Faraday's law of induction (or Maxwell's equation) then gives the electric field $\vec{E}(t)$ associated with the time varying magnetic induction:

$$\vec{\nabla}\times\vec{E}(\vec{r},t)=-\frac{\partial\vec{B}(\vec{r},t)}{\partial t}=-\mu_0 M_s\frac{\partial\hat{m}(\vec{r},t)}{\partial t} \quad . \tag{4}$$

Combining Equations (3) and (4), we get

$$\begin{aligned}\vec{\nabla}\times\vec{E}(\vec{r},t)=&\frac{\gamma\mu_0 M_s}{1+\alpha^2}\left(\hat{m}(\vec{r},t)\times\left[\vec{H}_{SOT}(\vec{r},t)+H_{shape}(\vec{r},t)\right]\right)\\&+\frac{\gamma\alpha\mu_0 M_s}{1+\alpha^2}\left[\hat{m}(\vec{r},t)\times\left(\hat{m}(\vec{r},t)\times\left[\vec{H}_{SOT}(\vec{r},t)+H_{shape}(\vec{r},t)\right]\right)\right]\end{aligned}. \tag{5}$$

Using the fact that $\hat{m}(\vec{r},t)\times\vec{H}_{shape}(\vec{r},t)\approx 0$ and also the fact that since cobalt is a low-damping material, we can ignore the Gilbert damping, we can simplify the above equation to

$$\vec{\nabla}\times\vec{E}(\vec{r},t)=\gamma\mu_0 M_s\left[\hat{m}(\vec{r},t)\times\vec{H}_{eff}^{SOT}(\vec{r},t)\right]. \tag{6}$$

Combining Equations (2) and (6), we get

$$\vec{\nabla}\times\vec{E}(\vec{r},t)=\frac{J(t)\hbar}{2ed}\gamma\hat{m}(\vec{r},t)\times\left(\eta_D\left[\hat{m}(\vec{r},t)\times\hat{n}\right]+\eta_F\hat{n}\right). \tag{7}$$

Next, using Stokes theorem in Equation (7) to relate a line integral to a surface integral, we obtain

$$
\begin{aligned}
&\oint \vec{E}(\vec{r},t)\bullet d\vec{l} = \int\left[\vec{\nabla}\times\vec{E}(\vec{r},t)\right]\bullet d\vec{S} \\
&= \frac{J(t)\gamma\hbar}{2ed}\int\left\{\hat{m}(\vec{r},t)\times\left(\eta_D\left[\hat{m}(\vec{r},t)\times\hat{n}\right]+\eta_F\hat{n}\right)\right\}\bullet d\vec{S} \\
&= \frac{J(t)\gamma\hbar}{2ed}\left[\eta_D\int\begin{matrix}\hat{m}(\vec{r},t)\times\left(\hat{m}(\vec{r},t)\times\hat{n}\right)\bullet d\vec{S} \\ +\eta_F\int\left(\hat{m}(\vec{r},t)\times\hat{n}\right)\bullet d\vec{S}\end{matrix}\right] \\
&= \frac{J(t)\gamma\hbar}{2ed}\eta_D\int\left[\hat{m}(\vec{r},t)\left(\underbrace{\hat{m}(\vec{r},t)\bullet\hat{n}}_{0}\right)-\hat{n}\left(\underbrace{\hat{m}(\vec{r},t)\bullet\hat{m}(\vec{r},t)}_{1}\right)\right]\bullet d\vec{S} \\
&+\frac{J(t)\gamma\hbar}{2ed}\eta_F\int\left(\hat{m}(\vec{r},t)\times\hat{n}\right)\bullet d\vec{S} \\
&= -\frac{J(t)\gamma\hbar}{2ed}\eta_D\int\hat{n}\bullet d\vec{S}+\frac{J(t)\gamma\hbar}{2ed}\eta_F\int\left(\hat{m}(\vec{r},t)\times\hat{n}\right)\bullet d\vec{S}.
\end{aligned}
\tag{8}
$$

where we made use of the fact that the magnetization lies in the plane of the nanomagnet. The electric field cannot have a tangential component on the surface of the nanomagnet since it is made of a conductor, but there can be a normal component. The electric field lines are shown in the figure below. Looking at this figure, it is obvious that the second term in the right-hand-side of Equation (8) dominates over the first because $d\vec{S}$ (which is normal to the loop area enclosed by the electric field like) tends to lie in the plane of the nanomagnet. Hence

$$
\oint \vec{E}(\vec{r},t)\bullet d\vec{l} \approx \frac{J(t)\gamma\hbar}{2ed}\eta_F\int\left(\hat{m}(\vec{r},t)\times\hat{n}\right)\bullet d\vec{S}. \tag{9}
$$

The Poynting vector $\vec{\mathbf{P}}$ associated with electromagnetic emission from the SHNA is given by

$$
\vec{\mathbf{P}} = \vec{E}\times\vec{H}_{SW} \quad , \tag{10}
$$

where $\vec{H}_{SW}$ is the amplitude of the spin wave. Fig. 6 of the main manuscript gives the highest amplitude (in the x-direction) as 0.1 $M_s = 10^5$ A/m where M_s is the saturation magnetization of Co $= 10^6$ A/m.

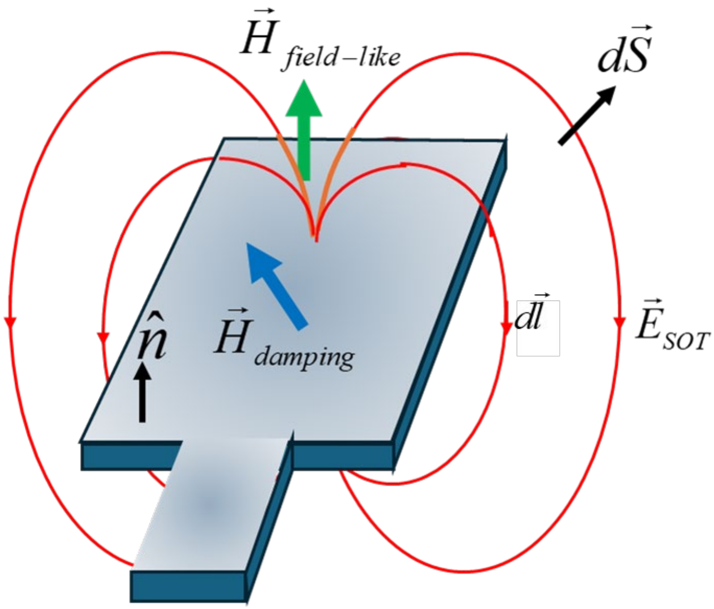

12.a Estimation of the electromagnetic power emitted by the SHNA

In the above figure, let us take the smallest electric field loop that will have a diameter of $W/2$, where W is the width of the nanomagnet = 200 nm. Since magnetic field does not exists outside the nanomagnet, we obtain from Equation (9) that the amplitude of the electric field will be roughly given by

$$E(\pi W/2) = \frac{J\gamma\hbar}{2ed}\eta_F Wd \Rightarrow E = \frac{J\gamma\hbar}{\pi e}\eta_F . \tag{11}$$

The power fed to the SHNA is P_{in} = 30.4 mW (see section 11). We have $P_{in} = I_{in}^2 R_{in}$, where R_{in} is the input resistance and I_{in} is the input current distributed among the 3000 Pt nanostrips in the sample. We measured R_{in} 9.9 ohms. Hence I_{in} = 55 mA and the current in any one nanostrip is $I_{in}/3000$ = 75.2 μA. The cross-sectional area of the Pt nanostrip is 300 nm $\times$5 nm and hence the current density amplitude J in Equation (11) is 4.6×10^{10} A/m². This yields that the electric field amplitude is $E = 2.1\,\eta_F$ V/m.

From the micromagnetic simulations in Fig. 6 of the main manuscript, the spin wave amplitude is 10^5 A/m. Hence, from Equation (10), the Poynting vector is $2.1\times10^5\eta_F$ W/m². Assuming that a fraction f of the 285,000 nanomagnets are radiating, the power P_t at the source would be $2.1\times10^5\eta_F\, f\times\underbrace{285{,}000\times240\times200\times10^{-18}}_{\text{nanomagnet array area}}\,\mathrm{W} = \eta_F\, f\ \times 2.87\mathrm{mW}$. Since we found that P_t is 255 μW (see Section 11), we conclude that the product $\eta_F\, f$ = 255 μW/2.87 mW = 0.088. This means that *at least* ~9% of the nanomagnets are radiating, which is very reasonable.

12.b The radiation is not due to classical oscillating magnetic dipoles.

One could think that since spin waves are periodically time varying magnetization, they could be viewed as analogous to classical oscillating magnetic dipoles that radiate electromagnetic waves. This picture would not be correct. We carry out a simple back-of-the-envelope calculation to show that the observed radiation cannot be due to classical oscillating magnetic dipoles.

In classical electromagnetic theory, the power radiated by a magnetic dipole is $P = \frac{\mu_0 \mathrm{M}^2 \omega^4}{6\pi c^3}$ where M is the magnetic moment of the dipole and ω is the angular frequency. The maximum value of M in our SHNA is $M_s \Omega$, where M_s is the saturation magnetization of Co (10^6 A/m) and Ω is the total nanomagnet volume which is 57×10^{-18} m^3. This yields the magnetic moment M as 57×10^{-12} A-m^2. At 3 GHz, the maximum radiated power from the oscillating magnetic dipoles is obtained from the above formula and turns out to be only about **0.1 pW**, which would not have been measurable and is several (more than 9) orders of magnitude smaller than what we measure. This tells us that the classical oscillating magnetic dipole picture cannot explain the observed intensity of radiation.

---

13. Data for a second sample – repeatability of the results

We fabricated and tested a second sample to check for repeatability of results. The SEM of the second sample is shown in Fig. S8 and the S_{11} plots for both real and control sample in the second set are shown in Fig. S9. The radiation patterns are plotted in Fig. S10. They were obtained under identical conditions as the first sample.

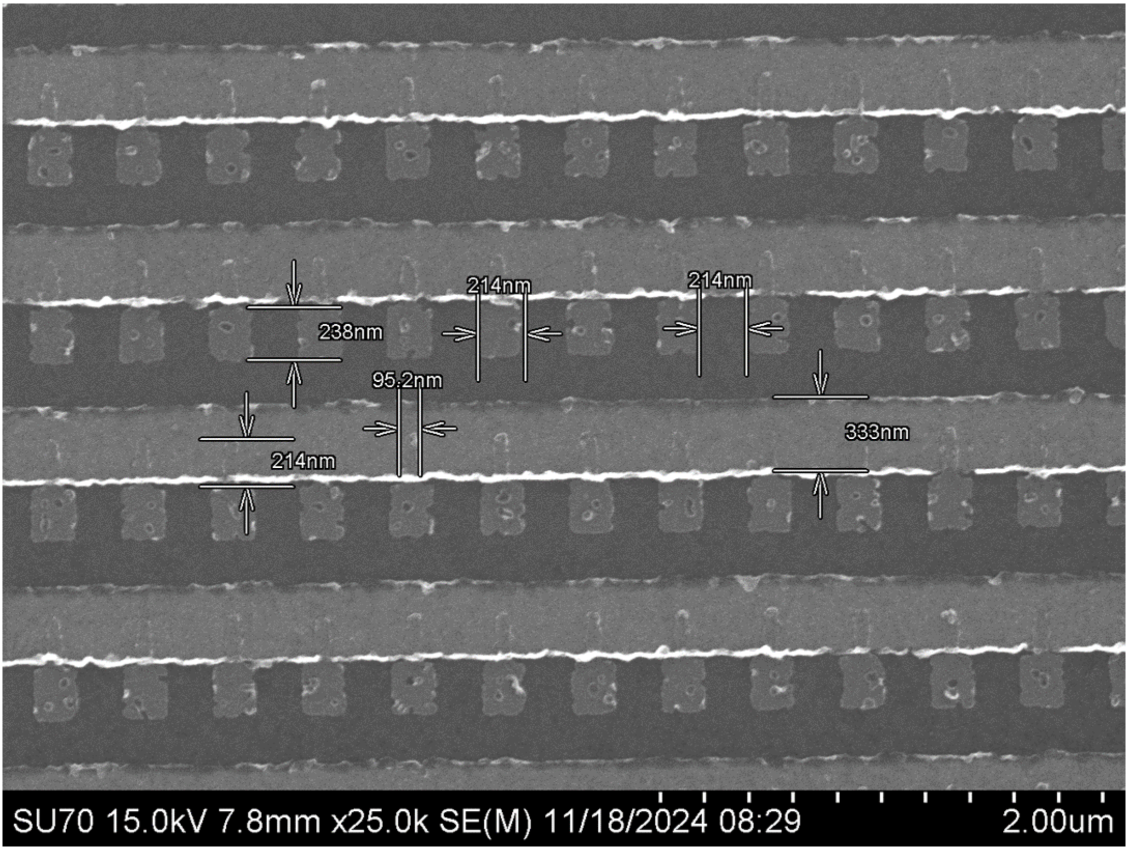

Fig. S8: Scanning electron micrograph of a second sample.

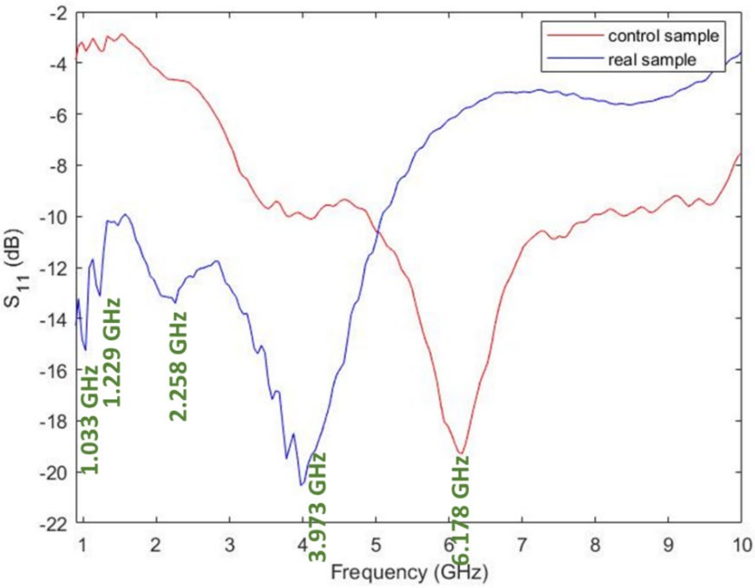

Fig. S9: S_{11} spectrum for the real and the control sample.

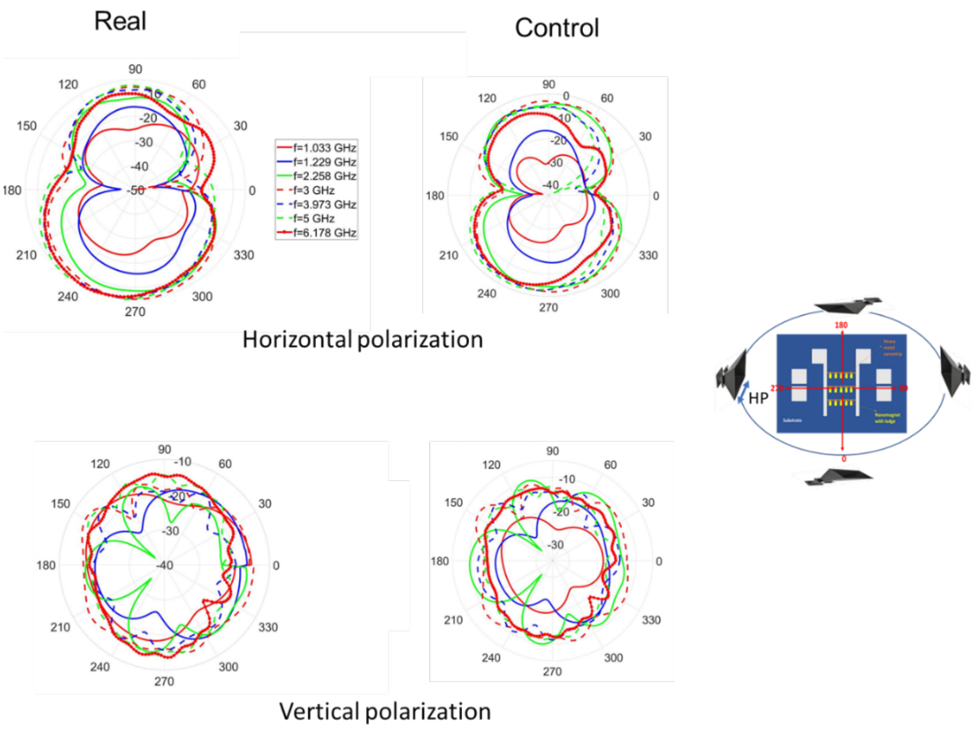

Fig. S10(a): Radiation patterns for the second sample (real and control) in the plane of the nanomagnets for both horizontal and vertical polarizations at different frequencies.

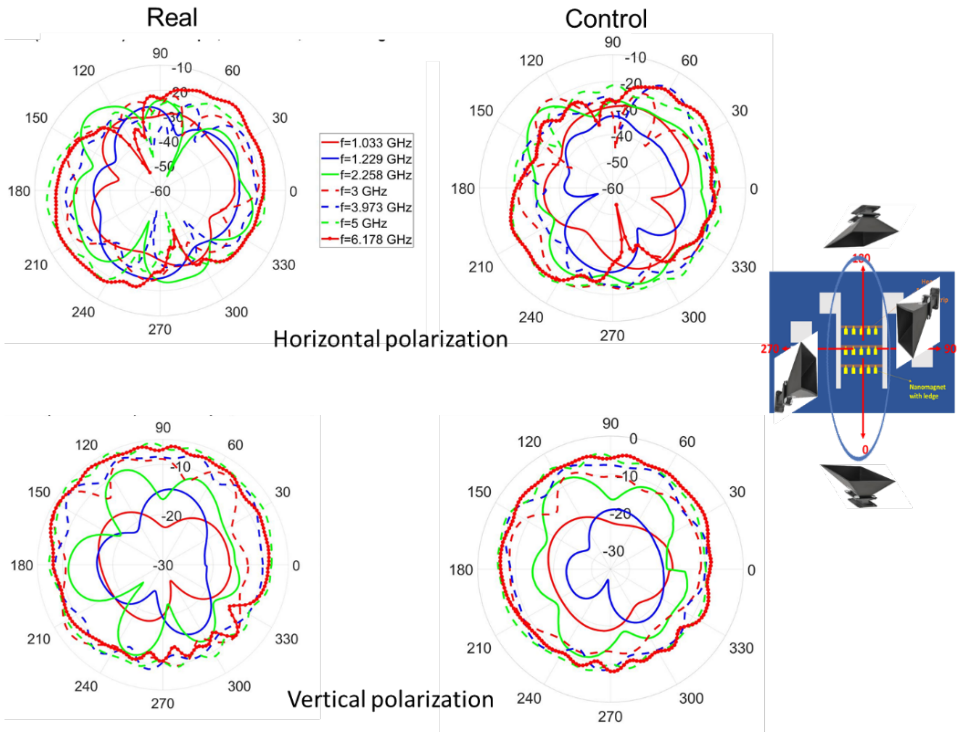

Fig. S10(b): Radiation patterns for the second sample (real and control) in a plane transverse to the plane of the nanomagnets for both horizontal and vertical polarizations at different frequencies.

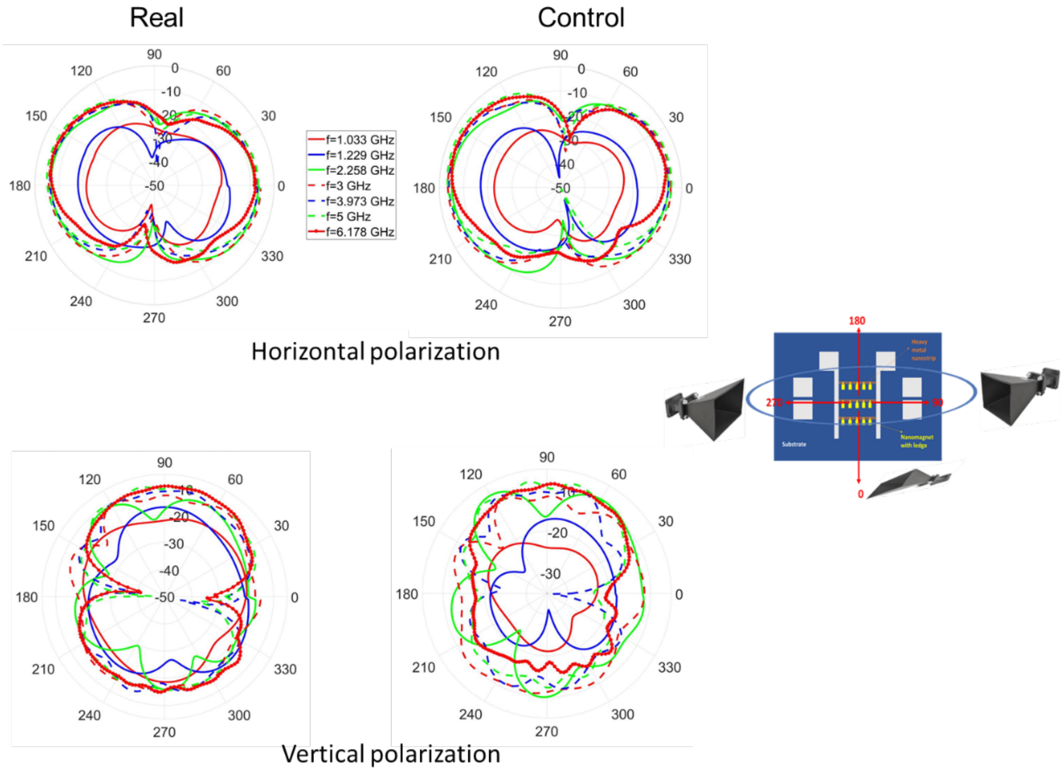

Fig. S10(c): Radiation patterns for the second sample (real and control) in the other plane transverse to the plane of the nanomagnets for both horizontal and vertical polarizations at different frequencies.

Once again, we find significant differences between the radiations from the real and the control sample in certain directions and certain frequencies, particularly in the two planes transverse to the plane of the nanomagnets. To highlight this difference, we have plotted the difference between the gains of the real and the control sample in dbi for all three planes. This is shown below in Fig. S11. This difference, however, should not be interpreted as the radiation pattern of the nanomagnets alone since we cannot get the radiation from the nanaomagnets by subtracting the radiation from the control sample from that of the real sample.

Looking at the left figure in the bottom row (horizontal polarization, plane transverse to the nanomagnet plane) we see that for a frequency of 5 GHz, the radiation intensity at the receiver is 30 db (1000×) higher for the real sample compared to the control sample in the 293^0 direction. This difference of 1000×is too large to accrue from spurious effects and can only be due to the fact that the nanomagnets are radiating and the sample is acting as a SHNA. Large differences between the radiation from the real sample and the control sample are also observed at other frequencies for other directions, which further confirm this observation.

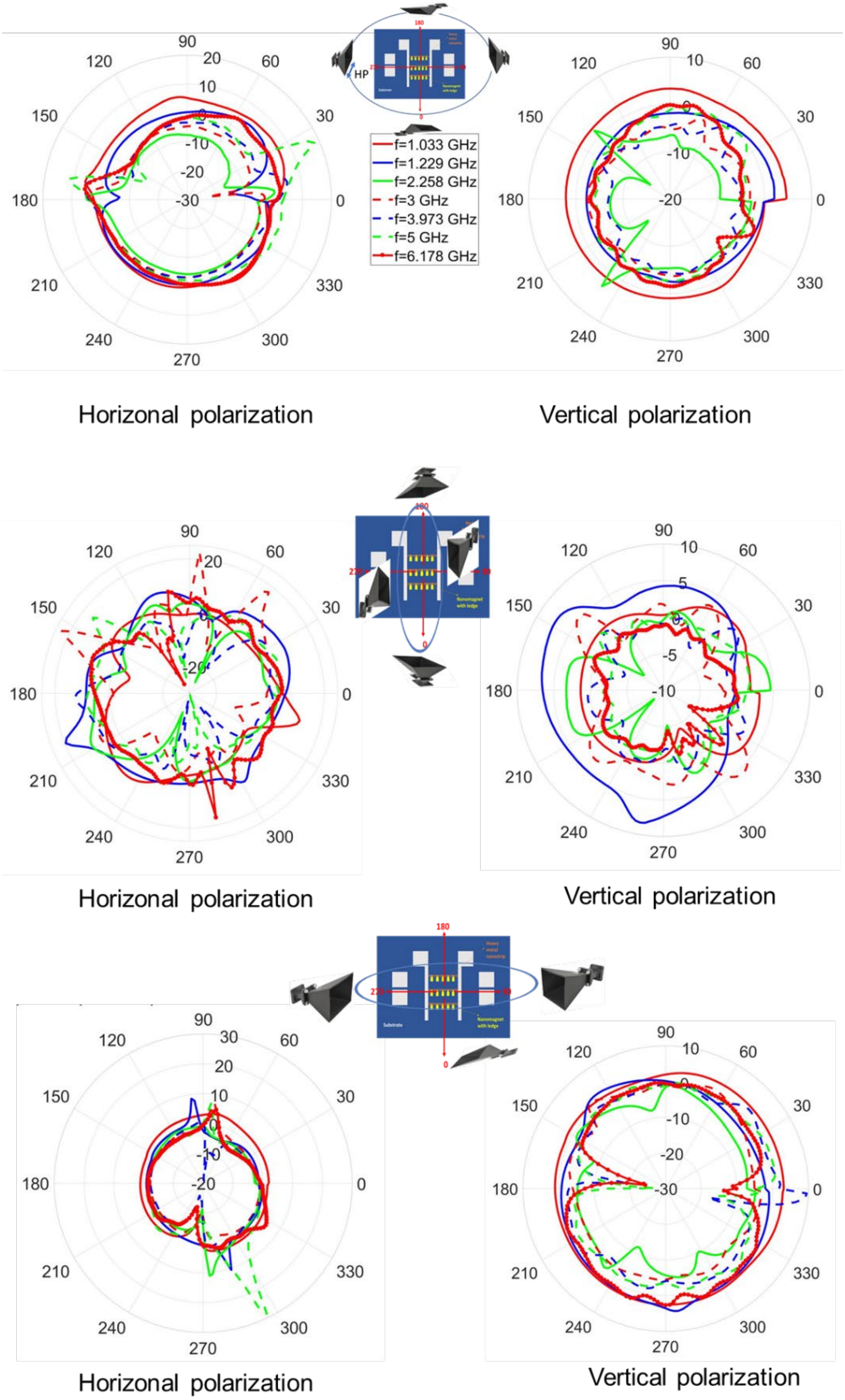

Fig. S11: Difference in the gain between the real and the control sample for radiation patterns in the plane of the nanomagnets and the two transverse planes plotted in dbi.

14. Are spin spin waves in the nanomagnets caused by spin-orbit torque or the time varying magnetic field caused by the passage of a time-varying current through the Pt nanostrips?

The fact that ac spin orbit torque excites spin waves in ferromagnets has been shown in [12] of the main paper, but that does not guarantee that it happens in our structures. Another possible origin of the spin waves is that the passage of an ac current through the Pt nanostrips makes them radiate electromagnetic waves, i.e., the nanostrips act as antennas and radiate an electromagnetic wave whose alternating magnetic field component is causing the spin waves. We show that this is *not possible* in our case.

The Pt nanostrips are 34 μm long and 300 nm wide. Therefore, each has a surface area A of 10.2×10^{-12} m^2. At 6 GHz (the highest frequency we consider), the electromagnetic wavelength λ is 5 cm. Hence, we are dealing with an *extreme sub-wavelength antenna* also known as an "electrically small antenna" (see [20,21] of the main paper). They are known to have very poor radiation efficiencies. The maximum radiation efficiency of such an antenna is on the order of $A/\lambda^2 = 4\times10^{-8}$ [20, 21]. At the highest power level of 16 dbm = 40 mW, which is pumped into the 3000 nanostrips that we have, each nanostrip receives a power of 13.3 μW on the average. Multiplying that by the radiation efficiency, we conclude that each nanostrip can radiate a maximum power of 5.32×10^{-13} watts. The total radiation due to all 3000 nanostrips is therefore 1.6 $\times10^{-9}$ watts, resulting in an areal power density of $1.6\times10^{-9}/(160\times10^{-12})$ = 10 W/m^2. Equating that to the pointing vector $\mathbf{E}\times\mathbf{H}$, where $\mathbf{E}$ is the electric field and $\mathbf{H}$ is the magnetic field in the radiated electromagnetic wave, we get $\eta|\mathbf{H}|^2$ = 10 W/m^2, where η (=$|\mathbf{E}|/|\mathbf{H}|$) is the free-space impedance of 377 ohms. This yields an order estimate of the magnetic field strength as 0.026 A/m which is much weaker than even the earth's magnetic field. Such a magnetic field is too tiny to excite spin waves of the amplitudes that we observe. Moreover, the SOT field (effective magnetic field due to SOT) that we estimated in Section 12 is 4,000 A/m, which is 150,000 times larger. Hence the spin-orbit torque is the dominant cause of the spin waves, not the magnetic field due to the ac current in the Pt nanowires.

15. Why is the radiation pattern anisotropic and difficult to reproduce in multiple samples?

Conventional wisdom has it that the radiation pattern of a "point source" will be isotropic and omnidirectional, i.e., the point source will radiate equally in all directions. The SHNA is much smaller than the wavelength and hence qualifies as a point source. Yet, its radiation pattern is very anisotropic. This oddity happens because the SHNA has *internal anisotropy* arising from the anisotropy of the spin wave patterns that form within the nanomagnets. We elucidate this below.

Consider first a single nanomagnet as shown in Fig. S12(a). From Fig. 6 of the main paper, we see that the amplitude of the spin wave oscillations in such a nanomagnets in the x-direction is 5 times that in the y-direction and 10 times that in the z-direction. This is caused by the odd shape

of the nanomagnets, *and it makes the spin wave pattern in every nanomagnet anisotropic.* Additionally, Fig. 6 shows that the phases of the *x*-, *y*- and *z*-components are also different. This means that

$$\vec{H}_{SW} = H_x e^{i\theta_x}\hat{x} + H_y e^{i\theta_y}\hat{y} + H_z e^{i\theta_z}\hat{z} \quad \left[H_x \neq H_y \neq H_z; \theta_x \neq \theta_y \neq \theta_z\right]$$

Consequently,

$$\vec{E} = E_x e^{i\phi_x}\hat{x} + E_y e^{i\phi_y}\hat{y} + E_z e^{i\phi_z}\hat{z} \quad \left[E_x \neq E_y \neq E_z; \phi_x \neq \phi_y \neq \phi_z\right]$$

Now consider two points α and β on the nanomagnets' plane (*x-y* plane) that are equidistant from the nanomagnet shown in Fig. S12(a). The former point α is on the *x*-axis and the latter (β) is on the *y*-axis. When the horn antenna is placed at point α as shown in Fig. S12(a), the horizontal polarization field it measures is proportional to $E_z(\alpha)$ and the vertical polarization field it measures is proportional to $E_y(\alpha)$. When the horn antenna is placed at point β, the horizontal polarization field it measures is proportional to $E_x(\beta)$ and the vertical polarization field it measures is proportional to $E_z(\beta)$. Since $E_x \neq E_y \neq E_z$, it is obvious that $E_z(\alpha) \neq E_x(\beta)$ and $E_y(\alpha) \neq E_z(\beta)$. Hence, the fields for either polarization measured at points α and β are not the same. That explains why the radiation patterns in Fig. 5 will be *anisotropic for both horizontal and vertical polarizations*.

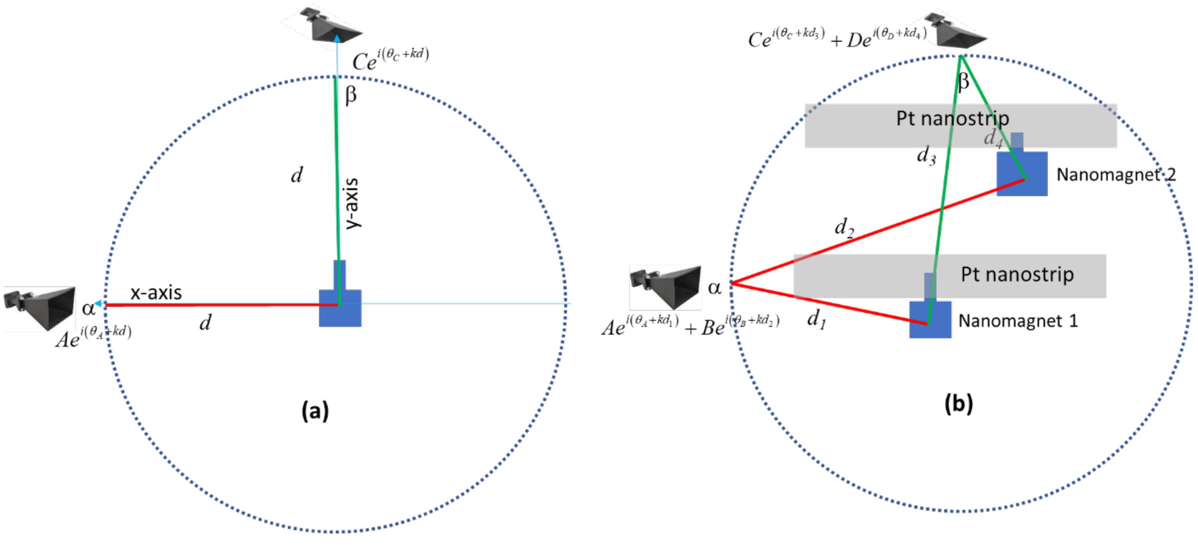

Fig. S12: Explanation as to why the radiation pattern is anisotropic in the *x-y* plane and very difficult to reproduce exactly from one sample to another Similar arguments will hold for the *x-z* and *y-z* planes.

We now explain why the radiation intensity from this seemingly point source will <u>not be equal</u> in all directions. Recall from Equation (10) that the Poynting vector is $\vec{\mathbf{P}} = \vec{E} \times \vec{H}_{SW}$ where $\vec{H}_{SW}$ is the magnetic field due to the spin wave. The intensity of radiation at point α is proportional to the Poynting vector's *x*-component which is given by $P_x = E_y H_z - E_z H_y$ while the intensity of

radiation at point β is proportional to the *y*-component of the Poynting vector $P_y = E_z H_x - E_x H_z$, where E_i and H_i are the *i*-th components of *E* and $\vec{H}_{SW}$. Since H_x is 10 times H_z and 5 times H_y owing to spin wave anisotropy, it is obvious from the above expressions that $P_x \neq P_y$. Hence, the electromagnetic intensity I_α at point α (which is proportional to P_x) *is different* from the electromagnetic intensity I_β at point β (which is proportional to P_y). This will also make the "amplitudes" of the radiation different, i.e., $A \neq C$ in Fig. S12(a). Similar arguments will hold for the *x-z* and *y-z* planes.

However, we do not have a single nanomagnet but have 285,000 nanomagnets and the received intensity the two points α and β on the circumference of a circle centered on the center of the nanomagnet array will depend on the interference between the beams emitted by all the 285,000 nanomagnets that reach α and β. (Actually, we estimated earlier that only about 9% of the nanomagnets radiate but that is not important for this discussion). To understand the effect of interference, consider two nanomagnets as shown in Fig. S12(b). The intensities at the two points α and β due to the interference of the beams radiated by these two nanomagnets is

$$I_\alpha = \left| A e^{i(\theta_A + kd_1)} + B e^{i(\theta_B + kd_2)} \right|^2 = A^2 + B^2 + 2AB\cos\left[\left(\theta_A - \theta_B\right) + k\left(d_1 - d_2\right)\right]$$

$$I_\beta = \left| C e^{i(\theta_C + kd_3)} + D e^{i(\theta_D + kd_4)} \right|^2 = C^2 + D^2 + 2CD\cos\left[\left(\theta_C - \theta_D\right) + k\left(d_3 - d_4\right)\right]$$

The spacing between the two nanomagnets is much smaller than the wavelength since this is an extreme sub-wavelength antenna. Hence $k\left(d_1 - d_2\right)$ and $k\left(d_3 - d_4\right)$ are both very small and can be neglected. This will make

$$I_\alpha \approx A^2 + B^2 + 2AB\cos\left(\theta_A - \theta_B\right)$$

$$I_\beta \approx C^2 + D^2 + 2CD\cos\left(\theta_C - \theta_D\right)$$

We had already stated earlier that spin wave anisotropy makes $A \neq C$. That same spin wave anisotropy will also make $B \neq D$, while the phases of the *x*-, *y*- and *z*-components of the spin wave being different (see Fig. 6 of the main paper), makes $\theta_A \neq \theta_C, \theta_B \neq \theta_D$. Hence, it is clear that the electromagnetic intensities at the two points α and β will be different, even in the presence of interference between beams emitted by multiple nanomagnets, which will make $I_\alpha \neq I_\beta$. Thus, the anisotropy of the spin wave pattern gives this "point source" radiator internal anisotropy, and that makes the radiation pattern also anisotropic.

Irreproducibility: Next, consider the corresponding nanomagnet pair in a *different sample*. For that sample to have the same radiation pattern as the first, we have to ensure $A = C, B = D, \theta_A = \theta_C, \theta_B = \theta_D$. That is impossible to ensure with the fabrication accuracy available in an academic lab. This is the reason why we cannot reproduce the radiation pattern exactly from one sample to another. This is why the radiation patterns of the two samples reported here are *not* identical. The "details" are different but the "effect" is the same in both samples.

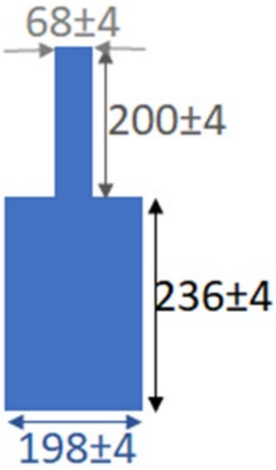

What makes device-to-device reproducibility impossible is the fact that the phases of the spin waves are especially sensitive to small variations in the nanomagnet shape and size, making it impossible to ensure that $\theta_A = \theta_C, \theta_B = \theta_D$. To highlight this point further, we show in Fig. S13 the phase profiles of three intrinsic modes of the magnonic crystal (M1, M2 and M3) calculated with MuMax3 and Dotmag in six different nanomagnets where we factored in small variations in the dimensions that were gleaned from the scanning electron micrograph of the actual sample. The figure above shows the maximum variations in the dimensions (in nm). In this case, the three intrinsic modes – which are natural modes of the magnonic crystal determined by the material, size, shape, spacing etc. of the nanomagnet array – had frequencies of 4.6 GHz, 6.6 GHz and 10.8 GHz. Note that for each of these three modes, the phase maps are very sensitive to small variations in the nanomagnet dimensions, which is why the color maps for phase are quite different in the six nanomagnets. This extreme sensitivity of the phase to small structural variations makes it impossible at this time to reproduce the radiation pattern from one sample to another.

16. Receiver antenna results for 1.5 GHz frequency

We obtained results when the excitation frequency was 1.5 GHz instead of 2.4 GHz. These results are shown in Fig. S14.

Unlike in the case of 2.4 GHz excitation, here the period and waveform of the input and output are the same, which raises the specter of the output signal being due to electromagnetic pickup. However, *there is a phase difference between the two*. The time taken by the electromagnetic wave to travel from the horn to the sample in this case will be $\Delta t = (6 \text{ in}/3\times10^8 \text{ m/s}) = 0.5$ ns and hence the phase shift between the input and output would have been $\varphi = 2\pi f \Delta t$ ($f = 1.5$ GHz) = 4.7 radians = 1.56 radians (modulo 2π), if it were electromagnetic pickup. The actual phase shift (modulo 2π) is about 3 radians, which does not match φ. Hence, it is most likely not due to direct electromagnetic pickup. In this case, we could not test the control sample and hence cannot confirm this independently.

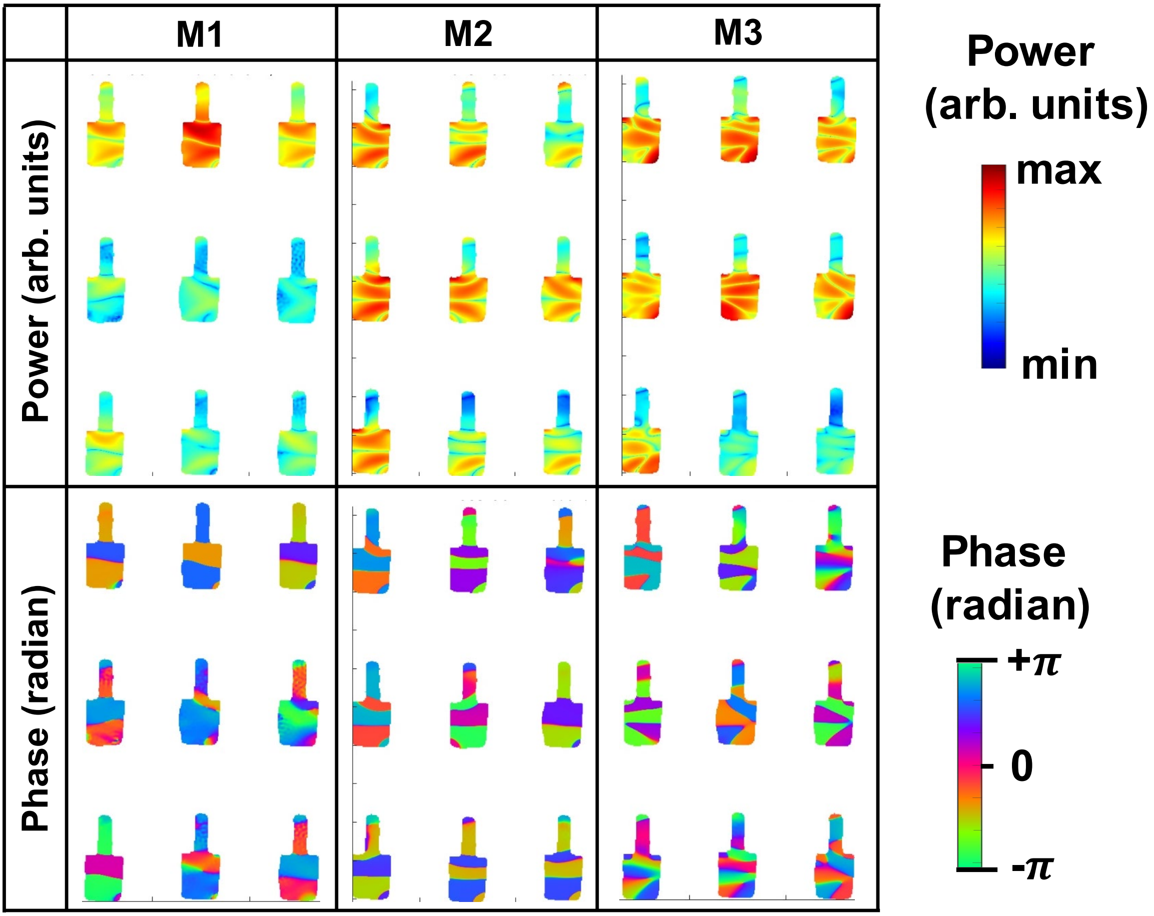

Figure S13: Simulated spatial distribution of power and phase profiles for the three SW modes in absence of bias magnetic field and ac-SOT (true intrinsic modes of the magnonic crystal). Color bars to interpret the power and phase profiles are presented on the right-hand side of the figure.

In Fig. S15, we show the Fourier transform of the received signal. Note that there are frequency components in the received signal at 750 MHz and 2.5 GHz which also are present in the output when the input signal frequency is 2.4 GHz. Since these are independent of the input signal frequency, they must correspond to intrinsic modes of the system. A major difference between EM excitations at 1.5 GHz and 2.4 GHz is that in the former case, the EM wave excites both intrinsic and extrinsic spin wave modes in the nanomagnets and hence the output voltage has frequency components at the frequencies of the intrinsic modes (750 MHz and 2.5 GHz) as well as the frequency of the EM wave (1.5 GHz). The latter is the frequency of the extrinsic mode spawned by the excitation. In fact, the signature of the extrinsic mode is dominant over those of the intrinsic modes in the output voltage. In contrast, the 2.4 GHz EM wave does not produce a 2.4 GHz frequency component in the output voltage but instead produces components at 750 MH and 2.5 GHz which are associated with intrinsic modes of the nanomagnet array. However, this does not mean that the 2.4 GHz excitation does not produce any extrinsic mode. It may or may not have produced one at 2.4 GHz, but if it did, it

may not be resolvable from the intrinsic mode at 2.5 GHz since these two frequencies are very close. But in the context of the receiving antenna, these discussions are somewhat academic. What matters is that the incident EM signal produces a voltage output, which is all that is necessary to implement the receiving function.

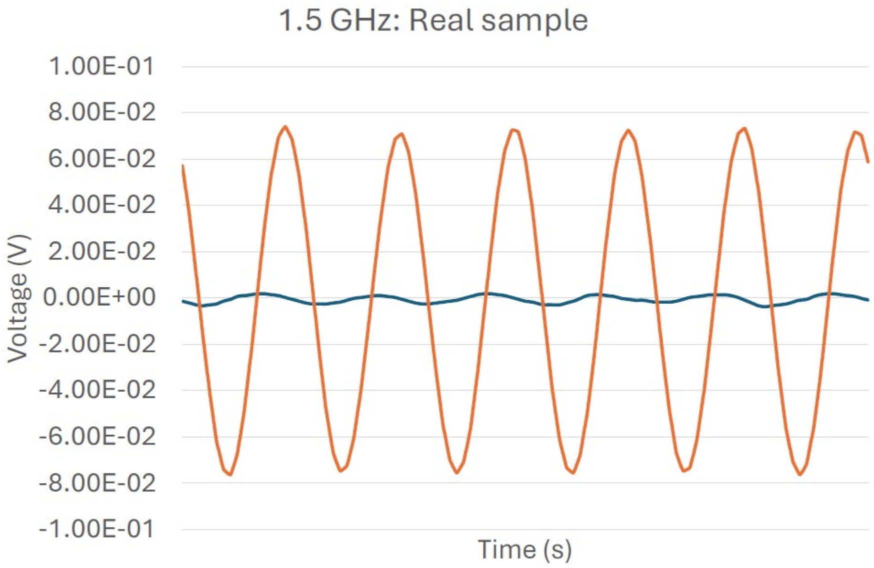

Figure S14: Digitized oscilloscope traces of the input signal fed to the horn antenna (in orange) and the output signal produced between the two output contact pads (in blue) in the real sample when the excitation frequency is 1.5 GHz and the horn-sample separation is 6 in. In this case, the amplitude of the input signal is roughly 15 times that of the output signal. There is a phase difference between the two which suggests that this is not due to direct electromagnetic pickup through the air.

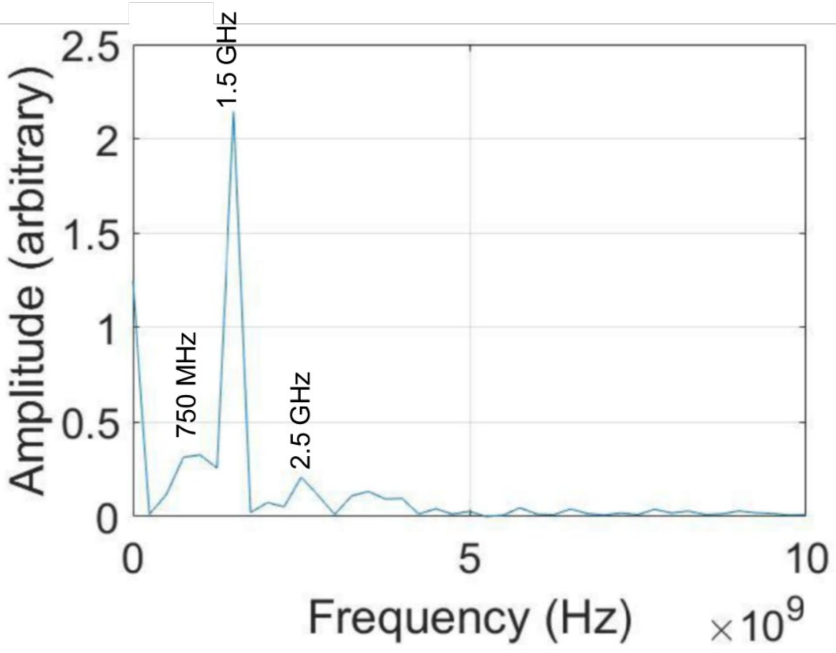

Figure S15: Fast Fourier transform of the received signal at 1.5 GHz excitation.

Receiver gain at 1.5 GHz

We can use Equation (1) from the main paper to calculate the receiver gain at 1.5 GHz:

$$\frac{P_r}{P_t} = \frac{V_{out}^2}{V_{in}^2} = (30)^2 = \frac{G_r G_t \lambda^2}{4\pi R^2}.$$

Using G_t = 9 dB = 8, λ = 20 cm, R = 6 in = 15.24 cm, we get G_r = 0.07 = -11.5 db.

References

1. W. A. Stutzman and G. A. Thiele, Antenna Theory and Design, (John Wiley & Sons, New York, 1981).
2. R. Fabiha, J. Lundquist, S. Majumder, E. Topsakal, A. Barman and S. Bandyopadhyay, "Spin Wave Electromagnetic Nano-Antenna Enabled by Tripartite Phonon-Magnon-Photon Coupling", *Adv. Sci*., **9**, 2104644 (2022).
3. N. Morrison, H. Taghinejad, J. Analytis and E. Y. Ma, "Coherent spin wave excitation with radio-frequency spin–orbit torque", *J. Appl. Phys*., **136**, 113901 (2024).
4. Y. J. Guo; X. H. Tang, K. F. Hou; W. X. Li and K. D. Xu, "A novel spin wave-based antenna using magnetic materials", 2017 IEEE International Symposium on Antennas and Propagation & USNC/URSI National Radio Science Meeting, San Diego, CA. **DOI:** 10.1109/APUSNCURSINRSM.2017.8072603
